# Proteomic signatures of *Staphylococcus aureus* biofilm maturation on orthopaedic implants

**DOI:** 10.1016/j.bioflm.2025.100287

**Published:** 2025-05-27

**Authors:** C. Kuik, C. de Boer, S.W.G. van Hoogstraten, K. Freulings, M. Honing, J.J.C. Arts, B. Cillero-Pastor

**Affiliations:** a Maastricht MultiModal Molecular Imaging Institute (M4i), Maastricht University, Maastricht, the Netherlands; b Department of Orthopaedic Surgery, Laboratory for Experimental Orthopaedics, CAPHRI, Maastricht University Medical Center, Maastricht, the Netherlands; c Department of Biomedical Engineering, Orthopaedic Biomechanics, Eindhoven University of Technology, Eindhoven, the Netherlands; d The MERLN Institute for Technology-Inspired Regenerative Medicine, Department of Cell Biology-Inspired Tissue Engineering, Maastricht University, the Netherlands

**Keywords:** Biofilm, Proteomics, Bacterial infection, AMR, Orthopaedic implants

## Abstract

Implant-associated infections pose a significant clinical challenge in the orthopaedic field, often leading to implant failure and revision surgeries. These infections are hard to treat, particularly due to the formation of bacterial biofilms. Orthopaedic implant surfaces feature varying roughness and compositions to optimise implant osseointegration and performance. Highly polished surfaces are used in articulating areas of high shear force to minimise wear particle formation, while rough or porous surfaces enhance implant and bone fixation. However, increased surface roughness or porosity can also promote bacterial adhesion and biofilm formation, potentially elevating the risk of chronic infections. In this study, an automated single-pot solid-phase enhanced sample preparation protocol (SP3) workflow was developed to investigate the differences in proteomic response of immature and mature *S. aureus* biofilms on titanium (Ti) surfaces with varying roughness (polished, corundum-blasted), and a plasma-sprayed microporous calcium phosphate coated surface (plasmapore), representing clinically relevant orthopaedic implants. Mature biofilms showed increased proteins related to toxin activity and the tricarboxylic acid (TCA) cycle, while immature biofilms had elevated proteins tied to binding, catalytic activities, and metabolism, suggesting surface topography influences early biofilm formation. This study highlights potential protein targets for novel antimicrobial therapies and suggests testing these as coatings on Ti surfaces, with the proteomics platform serving as a tool to evaluate bacterial and host responses.

## 1 Introduction

Titanium (Ti) alloys are a frequently used material for knee and hip replacements, as well as spine implants, due to their chemical and mechanical stability, high corrosion resistance, and excellent biocompatibility [1]. However, specific properties of Ti surfaces, such as the absorption of calcium, phosphate, and serum proteins, promote bacterial growth, biofilm formation, resulting in implant-associated infections [2,3]. This is the most significant concern following implant placement surgery, with approximately 2–5 % of implants failing prematurely [4]. It is estimated that biofilm-producing pathogens are responsible for approximately 65–80 % of all bacteria-associated infections and present a significant cause of biomaterial failures [5,6].

A biofilm is a natural state of bacteria in which the cells exist in sessile communities attached to a surface. Biofilms form in three stages, starting with the initial phase, where planktonic bacteria adhere to a surface through chemical and physical forces [7]. This is followed by the maturation stage, where microcolonies increase in size and thickness. The final stage is the dispersal phase, during which the biofilm is disrupted, allowing bacteria to migrate to other body sites. Bacteria in the biofilm exist in an enclosed multicellular community within an extracellular polymeric substance (EPS) comprised of water, polysaccharides, nucleic acids, proteins and lipids. The EPS functions as a protective barrier around the bacteria that shields them from the host's immune defence. Furthermore, it is accepted that bacteria in a biofilm are up to 5.000 times less susceptible to antibiotics than planktonic bacteria [8]. Most clinically isolated biofilm-forming pathogens from orthopaedic and trauma surgery implants are *Staphylococcus aureus (S. aureus), Staphylococcus epidermidis,* and *Pseudomonas aeruginosa* [9]. With *S. aureus* as the most frequently isolated pathogen, responsible for 19–29 % of all prosthetic joint-related infections [10,11].

Biomaterial surface characteristics, such as porosity and roughness, affect the implant integration with the surrounding tissue [12,13]. Previous studies demonstrated the effect of Ti surface roughness and topography on cell behaviour, such as promoting bone formation around the implant, known as osseointegration [2,14,15]. Consequently, the specific implant surface properties depend on factors such as its location in the body, mechanical loading, and intended function. Smooth, polished surfaces are typically utilised to minimise wear for smooth articulation, as needed at the neck of a hip implant, for example. For the femoral stem of hip implants, however, surfaces with high surface roughness are preferred for optimal integration into bone tissue. While high surface roughness promotes implant integration, it is also associated with increased bacterial adhesion and biofilm formation due to more availability of binding sites and improved protection of bacterial cells [14,16]. However, the underlying molecular mechanisms of *S. aureus* biofilm in response to implant roughness and chemical composition remain largely unexplored. Investigating the response of bacteria concerning biomaterial characteristics will yield valuable insights for improved antimicrobial technologies. These insights can help the development of novel antimicrobial surface therapies, targeting crucial pathways for better implant integration in parallel to reduced biofilm formation.

Proteins play a vital role in the biological processes of cells and are the primary targets for many drugs [17,18]. Proteomics methodologies allow for understanding the essential proteins modulating bacterial response [19]. Previously, proteomics has been applied to investigate the protein changes related to antibiotic resistance, physiological processes, and environmental conditions [[20], [21], [22]]. Furthermore, proteomics studies confirmed a significant change in the *S. aureus* biofilm proteome correlated to biofilm ageing. Multiple pathways were found to be modulated, and numerous proteins involved in the central metabolism pathways were observed in the early-phase biofilms [20].

Protein investigation of bacterial cells and bacterial biofilm communities faces technical challenges regarding sufficient protein recovery and a high background of the exopolysaccharides in the extracellular matrix (ECM) [23]. Recently, multiple sample preparation protocols have been applied to circumvent these challenges. One method to enhance protein coverage is by optimising cell lysis and protein recovery using detergents [24]. However, most ionic detergents are not compatible with mass spectrometry-based (MS) analysis. N-dodecyl-β-D-maltoside (DDM) has been demonstrated as a non-ionic surfactant that does not cause ion suppression during the MS analysis while increasing the solubilization of membrane proteins [24]. Furthermore, sodium 3-[(2-methyl-2-undecyl-1,3-dioxolan-4-yl)methoxy]-1-propanesulfonate, also known as Rapi*gest*™ is an anionic detergent that, among others, improves protein unfolding and solubilization by acting as a mild protein denaturing agent and is commonly used in proteomics pipelines [25].

An improved sample preparation proteomics workflow can be achieved by using a single-pot solid-phase enhanced sample preparation protocol (SP3), previously introduced by Hughes et al. [26,27] SP3 uses magnetic beads for protein cleanup and enzymatic digestion, enabling the use of detergents and buffers for protein isolation. The protocol has improved the protein detection of various planktonic bacteria, including *S. aureus* [28]. Furthermore, the SP3 protocol has been compared to in-solution digestion, S-trap, and gel digestion, clearly exhibiting an increase in detected peptides of various planktonic bacteria when using SP3 [29,30]. However, so far, no studies have investigated the advantages of this method when analysing bacteria in biofilms. Here, an automated SP3 workflow was employed for increased reproducibility.

In this study, we developed and applied a label-free proteomics workflow, including isolation, digestion and cleanup in an automated pipeline, to investigate the *S. aureus* proteome response of immature and mature biofilms to different clinical Ti implants by using LC-MS data-independent acquisition (DIA). The investigated materials consisted of polished Ti, corundum-blasted Ti, and Ticoated with a porous calcium-phosphate layer. First, the cell lysis and protein recovery were optimised by investigating two MS-compatible detergents, sodium 3-[(2-methyl-2-undecyl-1,3-dioxolan-4-yl)methoxy]-1-propanesulfonate and DDM. Secondly, the efficiency and robustness of the digestion protocol were evaluated by employing an SP3 pipeline.

Study outcomes will be valuable in understanding biofilm formation and maturation on commonly used surfaces and screening target proteins to prevent or treat biofilm formation.

## 2 Methods

### 2.1 Test samples

Biofilm formation was assessed on sterile Ti alloy (Ti–6Al-4 V) discs (Ø 5 mm × 14 mm; B. Braun, Tuttlingen, Germany) discs. The investigated surfaces consisted of polished Ti, corundum-blasted Ti, and Ti coated with a porous calcium-phosphate layer with various topography roughness profiles and compositions: highly polished (Ra <0.2 μm), corundum blasted (Ra 3.5–5.5 μm), and plasma-sprayed microporous coated (Plasmapore®, Aesculap AG, Tuttlingen, Germany) with a pore size of 50–200 μm and a microporosity of 35 %. The coated items were packed individually and gamma sterilised (BBF Sterilisationsservice GmbH, Kernen-Rommelshausen, Germany). For each disc type, 14 discs were included per experiment.

### 2.2 Bacterial culture

*S. aureus* ATCC 25923 (American Type Culture Collection), stored in glycerol at −80 °C, were streaked on blood agar plates and grown overnight at 37 °C. Two colony-forming units (CFU) were inoculated in 5 ml tryptic soy broth (TSB; Sigma-Aldrich, St Louis, MO, USA, 22092) and incubated overnight at 37 °C, 200 rpm. The overnight culture was washed with phosphate-buffered saline (PBS) and diluted with TSB to a target concentration of 1 × 10^6^ CFU/ml.

### 2.3 Biofilm proliferation

The test discs were placed in a 24-well plate, and 1 ml of the bacterial suspension (10^6^ CFU/ml) was added to each disc. The discs were incubated for 4 h at 37 °C for bacterial attachment. The discs were then gently washed in 2 ml PBS to wash off non-adherent bacteria and placed in fresh TSB (2 ml). Biofilms were cultured statically at 37 °C for two days (immature biofilm) and seven days (mature biofilm), with media refreshment every three days. After incubation, the discs were gently washed in 2 ml PBS to remove non-adherent bacteria and processed as described below. The experiment was performed in triplicate.

### 2.4 Biofilm quantification – CFU counts

Following biofilm formation, the viable cells were quantified for the immature and mature biofilms grown on various Ti topographies. Three discs were used to quantify CFU after culture for each disc type. The discs were placed in 50 ml tubes containing 5 ml sterile PBS and underwent 5-min water bath sonication (Branson 2210 sonicator, Branson Ultrasonics Co., Ltd, Danbury, CT, USA) and 1-s vortex to detach the cells from the Ti surface. The sonicate suspension was serially diluted, and 100 μl was plated on BD™ Columbia Agar with 5 % Sheep Blood, and agar plates were incubated at 37 °C for 24 h, after which the CFU were counted.

### 2.5 Biofilm quantification – biomass staining

To quantify the biomass of biofilm formed, discs were stained using safranin. Four discs per surface type were included for the staining. Each disc was rinsed twice with 2 ml Milli-Q water. The biofilms were fixed with 2 ml 10 % formalin (VWR, Radnor, PA, USA, 2090.368) for 10 min and rinsed three times with 2 ml Milli-Q water. Surfaces were air-dried for 30 min before being stained with 2 ml 0.1 % safranin for 10 min. Excess stain was washed off the discs with 2 ml Milli-Q water 3 times. The discs were air-dried for 30 min, and the safranin was solubilised with 1 ml of 33 % glacial acetic acid (VWR, 20102.292) for 30 min. The release-trapped dye was pipetted into a 96-wells plate using four technical replicates per sample, and the absorbance was measured using a Microplate Reader (Thermo Scientific Multiskan FC) at 540 nm.

### 2.6 Biofilm morphology using scanning electron microscopy

The biofilms were imaged using scanning electron microscopy (SEM) to visualise the morphology of the immature 2-day and mature 7-day biofilm grown on orthopaedic-relevant materials. Two discs per surface type were included for SEM imaging. Biofilms were grown as described above, washed with PBS, and fixed with 2.5 % glutaraldehyde (Sigma-Aldrich, G5882) in 0.1 M phosphate buffer (PB) at 4 °C. Briefly, the Ti discs were washed three times for 15 min with PB, fixed with 1 % osmium tetroxide 0.1 M PB for 1 h, and washed three times for 15 min with PB. The samples were dehydrated for 30 min in 70 %, 90 %, and 100 % ethanol and hexamethyldisilazane (Sigma-Aldrich, 440191) twice for 30 min. Samples were dried and mounted on specimen stubs with silver glue before sputter coating with 5 nm carbon using a Leica EM ACE600. The samples were examined using a JEOL SEM (JSM-IT200) at a voltage of 10 kV. Per sample, images were acquired at three different locations.

### 2.7 Protein isolation and cell lysis

Eight discs per disc type were used for proteomic analysis. The biofilms were grown and sonicated as described above. For each sample, the sonicate solution containing the *S. aureus* biofilm was centrifuged at 3500×*g* for 10 min to spin down and separate the bacterial cells from the PBS. Pellets from the eight individual biofilm samples were collected and treated with 30 μL of cold ammonium bicarbonate buffer 50 mM (ABC) (Sigma-Aldrich, Eindhoven, The Netherlands) to remove the excess PBS after additional centrifugation at 3500×*g* for 10 min. Next, the sample pellets were dissolved in 100 μL of Urea lysis buffer (5 M Urea (GE Healthcare, Eindhoven, The Netherlands), 50 mM ABC buffer) or 90 μL Urea lysis buffer containing either 9.9 μL of 0.1 % DDM (Sigma-Aldrich, Eindhoven, The Netherlands) or 9.9 μL of 0.1 % Rapi*gest*™ (Waters, Milford, Massachusetts, United States).

The samples underwent bead-beating and three freeze−thaw cycles to promote cell lysis and protein extraction by adding glass beads (1 mm) (Biospec, USA, Bartlesville). The samples were exposed to three cycles of shaking at 6500 rpm for 15 s, followed by 20 s of interruption using the Precellys Evolution Touch Homogenizer (Bertin Technologies, France). Each freeze-thaw cycle involved freezing the samples on dry ice, followed by 1 min of sonication. The samples were mixed on a vortex between cycles to ensure thorough mixing. Afterwards, the protein-containing supernatant was collected by centrifugation at 15000 rpm at 4 °C. Protein quantification was conducted using a Bradford assay (Bio-Rad GmbH, Basel, Switzerland). Finally, all samples were stored at −80 °C until further analysis.

### 2.8 Protein digestion

42 μg of protein in 50 μL of buffer, underwent digestion with the Agilent Bravo automated liquid handling platform. Employing the SP3 protocol, proteins were bound to paramagnetic beads while solvents were effectively washed away. Initially, proteins were reduced by adding 10 μL of dithiothreitol (DTT) at a concentration of 634.5 mM, followed by a 45-min incubation at room temperature. Alkylation was subsequently carried out with the addition of 5 μL of iodoacetamide (IAM) at a concentration of 1 M, followed by another 45-min incubation at room temperature. After, Sera-Mag™ Carboxylate-Modified Magnetic Speedbeads were introduced to the sample at a protein-to-bead ratio of 1:10, along with 50 % v/v acetonitrile (ACN) to enhance protein binding. After binding, the beads underwent three washes with ACN. For the digestion step, a Promega Trypsin/Lys-C mix, diluted in 50 mM ABC, was utilised at a protein-to-enzyme ratio 25:1. Simultaneously, 5 M urea was added to induce protein unfolding. After 2 h of incubation at 37 °C, the sample was diluted with 50 mM ABC to reduce the urea concentration to below 1 M, activating trypsin. After an overnight incubation at 37 °C, digestion was terminated by adding 5 % trifluoroacetic acid (TFA) to a final concentration of 1 % v/v. The beads were separated from the sample using an external magnetic rack and subjected to sonication in 2 % dimethyl sulfoxide (DMSO) for 3 min to facilitate the release of any remaining peptides bound to the beads. The remaining liquid was then separated from the beads and combined with the sample.

To compare the three digestion protocols, the in-solution digestion was performed manually or on the Agilent Bravo automated liquid handling platform. 50 μg of protein in 50 μL of urea buffer underwent reduction and alkylation with DTT and IAM to a final concentration of 20 mM and 40 mM, respectively. Each agent was introduced and incubated for 45 min at room temperature. An additional reduction step was performed by adding DTT to a final concentration of 38 mM; this solution was incubated for 45 min at room temperature. For protein digestion, trypsin/LysC was introduced in a ratio of 1:25 and statically incubated for 2 h at 37 °C. After incubation, 200 μL of 50 mM ABC was added to reduce the urea concentration. This mixture was incubated overnight at 37 °C. The digestion was stopped by introducing 30 μL of 20 % ACN and 10 % FA to the sample. Finally, the samples were centrifuged at 15,000×*g* for 30 min at 4 °C, and the resulting supernatant was collected and stored at −20 °C until further analysis.

### 2.9 Liquid-chromatography-based peptide separation

1 μL of peptide at a concentration of 0.35 μg/μL was injected in duplicate on an Acclaim PepMap C18 analytical column (15 cm, ID 75 μm, 3 μm) (Thermo Fisher Scientific, Waltham, USA) coupled to a Thermo Fisher Scientific Dionex Ultimate 3000 Rapid Separation ultrahigh-performance liquid chromatography (UHPLC) system (Thermo Fisher Scientific, Waltham, USA). The peptides were analysed in DIA mode. Before separation on the analytical column, the samples were trapped on a C18 column for desalting. The peptides were separated on the analytical column using a 30-min gradient. During this gradient, the mobile phase B (acetonitrile with 0.1 % formic acid) increased from 4 % to 32 % at a 300 nL/min flow rate. Mobile phase A contained water with 0.1 % formic acid.

### 2.10 Tandem mass spectrometry acquisition

After LC separation, peptides were analysed on an Orbitrap MS Q-Exactive instrument (Thermo Fisher Scientific, Waltham, USA) equipped with a Proxeon nanoelectrospray Flex ion source (Thermo Fisher Scientific, Waltham, USA). Before analysis, the system was externally calibrated with a Pierce LTQ Velos ESI source in positive ion mode, using a positive ion calibration solution (Thermo Fisher Scientific, Waltham, USA). In DIA mode, the settings were adapted from Pino et al. [31] In brief, the instrument acquired a 25 × 24 *m/z* precursor isolation window at a mass range of 400–1000 *m/z* and a resolution of 30,000 using a staggered window pattern. Precursor spectra with a mass range between 385 and 1015 *m/z* at a resolution of 60,000 were interspersed every 25 MS/MS spectra.

### 2.11 Data processing

Raw DIA data files were imported into DIA-NN (version 1.8.1, https://github.com/vdemichev/DiaNN) for a predicted library database search. Proteins were identified by matching the ions against the Swiss-Prot *S. aureus* database, TaxID 1280 [32]. This database was downloaded from UniProt (https://www.uniprot.org/) and accessed in March 2024. Trypsin/P was used for this database search, allowing one missed cleavage, carbamidomethylation of Cysteine modification and oxidation of Methionine of the Protein N-term. A peptide length between 7 and 30 amino acids was allowed with a precursor charge between 1 and 4. The precursor *m/z* ranged from 385 to 1015 with an false discovery rate (FDR) of 0.1 %, and fragment ions ranged from 200 to 1800.

The processed data was analysed with Perseus (version 2.0.7.0) [33]. Before the statistical testing, the data was log (2) transformed and filtered on four valid values per group, and missing values were replaced from the normal distribution (width 0.3 and downshift 1.8). T-tests were carried out using a both-sided student t-test with an S0 of 0, and Benjamini-Hochberg was used for truncation with an FDR of 0.05. Proteins with a student t-test difference above 0.58 or below −0.58 (>1.5 fold change) and q-value <0.05 were considered differentially expressed. Multiple sample tests were carried out to compare the three different surface roughnesses, performed with ANOVA testing, using an S0 of 0, and Benjamini-Hochberg, with an FDR of 0.05, was used for truncation. The ANOVA significant proteins were visualised by hierarchical clustering after z-score normalisation based on the row's mean data. Volcano plots were processed with a both-sided *t*-test, an FDR of 0.05, and a randomisation of 250.

Protein enrichment analysis was performed by String (version 12.0, https://string-db.org/) using *S. aureus* as an organism. Pathways were considered relevant when the FDR was below 0.05, strength was above 0.01, and the minimum count in the network was 2. String functional enrichments were filtered based on gene ontology (GO) terms, including GO biological processes, GO molecular functions, and GO cellular components.

**Fig. 1:**
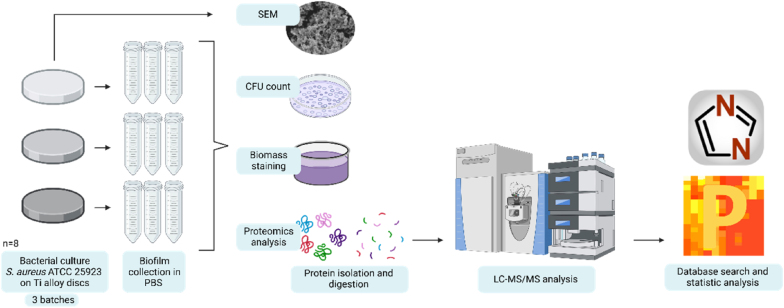
Methodological workflow used to study the influence of three Ti implant materials on *S. aureus* biofilm. Created with BioRender.com.

A detailed workflow used for this study is summarised in Fig. 1.

## 3 Results and discussion

### 3.1 Optimisation of proteomics workflow

**Fig. 2:**
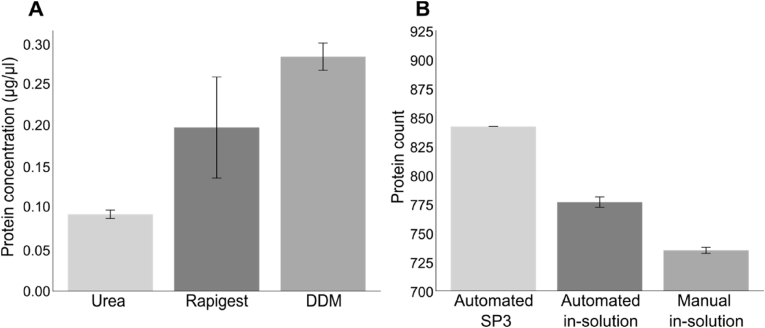
**Protein recovery and protein count of bacterial cells.** A) Protein concentration after cell lysis of planktonic bacterial cells using urea, urea with Rapigest™ or urea and DDM. B) Protein counts of planktonic bacteria after manual in solution, automated in solution, and automated-SP3 digestion.

Bacterial strains, including *S. aureus*, can form biofilms on Ti implant surfaces, causing complex implant-associated infections. The proteome response of these biofilms to different surface topographies and compositions was investigated during the biofilm maturation of *S. aureus*. However, studying the proteome of bacteria within a biofilm can be challenging due to the rigid cell wall of gram-positive bacteria and the composition of the ECM. We first evaluated the efficiency of DDM and Rapigest in urea buffer extracting proteins from a seven-day biofilm and planktonic bacteria. 0.1 % DDM improved protein extraction in planktonic bacteria as well as in biofilms (Fig. 2A). However, the increase in protein recovery from seven-day biofilm (Fig. S1A) samples was not as pronounced as the difference observed between extraction methods for planktonic bacteria (Fig. 2A).

Next, three workflows were evaluated to improve the efficiency of protein digestion from biofilm proteins: manual in-solution digestion, automated in-solution digestion and automated SP3 digestion. The SP3 digestion protocols used magnetic beads for on-bead digestion. This study presents the first comparison of manual and automation of sample preparation for proteomic biofilm analysis. The number of identified proteins in planktonic and biofilm samples is visualised in Fig. 2B. An increase in recovered proteins was observed with the automated protocols compared to manual digestion. Furthermore, the automated protocols experienced a lower standard deviation, resulting in higher reproducibility. Reproducibility is an essential factor when working with clinical applications [34,35]. Comparing the automated protocols, both in-solution and SP3 methods yielded the same number of identified proteins for the biofilm samples, as demonstrated in Fig. S1B. However, due to the on-bead digestion, the SP3 protocol offers more flexibility in using non-MS-compatible detergents or chemicals for cell lysis and protein recovery without requiring additional cleanup steps [36].

### 3.2 The effect of surface topography on biofilm growth

The optimised LC-MS-based proteomics protocol using DDM for cell lysis and the SP3 digestion method was used to investigate the differences in protein abundance in *S. aureus* biofilms over time in different Ti surface types: highly polished, corundum-blasted, or plasmapore-coated surfaces. SEM images of the three surfaces are displayed in Fig. S1.

**Fig. 3:**
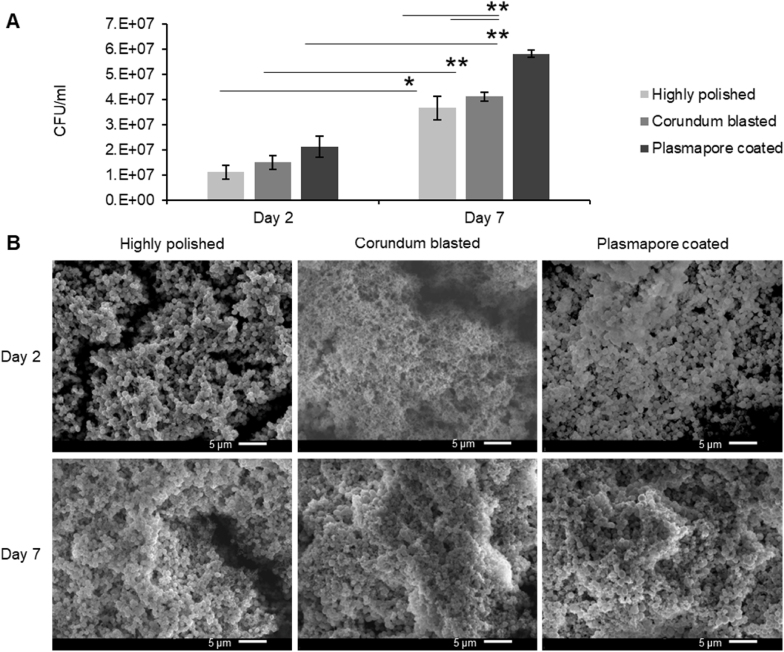
**Comparison of biofilm grown on various Ti topographies.***S. aureus* ATCC 25923 2-day and 7-day biofilm grown on highly polished, corundum blasted and plasmapore-coated Ti discs. A) Log scale of total CFUs of 2-day and 7-day biofilm extracted from the various topographical Ti are displayed—three independent experiments containing three technical replicates. Error bars indicate SEM. ∗∗p < 0.005 and ∗ p < 0.05 with *t*-test. B) Representative SEM images of 2-day and 7-day biofilm grown on various Ti topographies.

The biofilms were grown for two days or seven days to represent an immature and mature biofilm, respectively, based on the obtained CFU, SEM data, and literature [[37], [38], [39], [40]]. The average CFU of the biofilm extracted from the Ti discs is represented in Fig. 3A. As previously reported, the mature biofilm demonstrated a significant increase in bacterial growth and mass [20]. Furthermore, CFU counts were increased between the surface topographies, with the highest CFU levels observed for biofilm grown on plasmapore-coated material and the lowest for biofilm grown on polished material (Fig. 3A). Safranin staining was performed to investigate the correlation between bacterial growth and biofilm formation on the three surfaces (Fig. S2). The results demonstrated a correlation between bacterial growth and the increase in biofilm mass.

### 3.3 Protein patterns at different biofilm growth stages

**Fig. 4:**
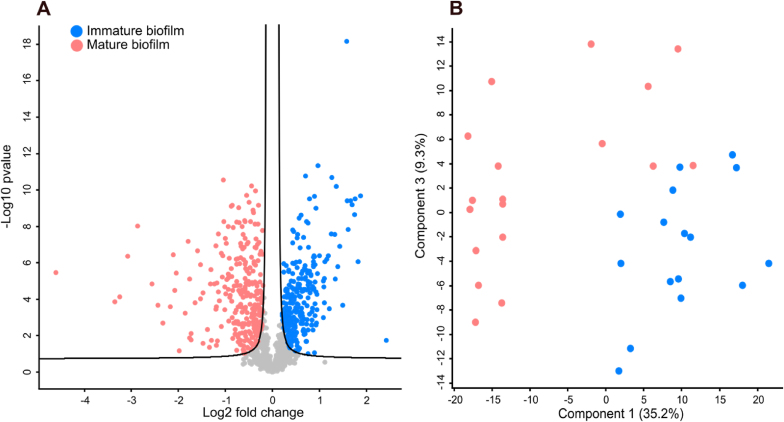
**Vulcano plot and PCA plot of immature and mature biofilm data.** A) The Vulcano plot acquired by Perseus displays upregulated proteins mature in pink and upregulated proteins immature in blue. B) PCA plot of component 1 and component 3. Mature biofilm samples are visualised in pink, and immature biofilm samples are visualised in blue.

The optimised extraction protocol was used for protein analysis, and 1151 protein groups were successfully identified and quantified. Proteins were further analysed across the growth stages of the biofilm. Differences between the two time points were investigated independently of the surface type. This resulted in an upregulation of 120 proteins on day two and an upregulation of 121 proteins at day seven (Fig. 4A, Table S1). Fig. 4B represents the separation of the immature and mature biofilms in a principal component analysis (PCA) plot. Proteins with the highest upregulation rates in immature biofilm included the extracellular matrix-binding Protein (ebh), among others. Ebh is a crucial protein for the initial stage of biofilm formation and is involved in pathogenesis, specifically fibronectin binding and intercellular adhesion [41,42]. Furthermore, Ornithine carbamoyltransferase, also highly expressed in the immature biofilm, is a metabolic enzyme facilitating arginine biosynthesis [43]. Both proteins are essential in biofilm growth and development.

On the contrary, the mature biofilm demonstrates an upregulation of two phenol-soluble modulins [44]. These proteins are connected to the structuring of the biofilm and the distribution of biofilm-associated infections. Delta-hemolysin was also upregulated at seven days, previously described as a protein involved in biofilm dispersal and formation. A decrease in delta-hemolysin is related to a reduction in biofilm dispersal and an increase in biofilm formation [45]. Therefore, upregulation of this protein will increase biofilm dispersal, characteristic of a mature biofilm.

**Fig. 5:**
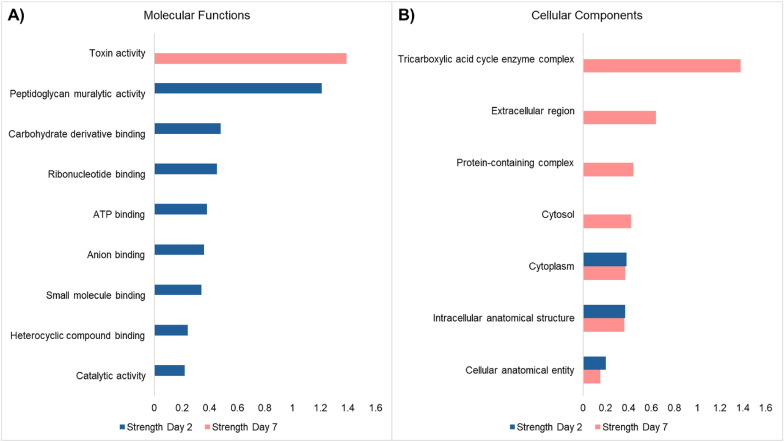
**Molecular and cellular functions upregulated per biofilm growth state.** GO molecular functions and cellular components are upregulated in the immature biofilm and are visualised in the left panel. GO molecular and cellular functions are upregulated in the mature biofilm as displayed in the right panel.

Pathway analysis demonstrated that the upregulated proteins are involved in multiple molecular and cellular GO processes (Fig. 5). These molecular processes were investigated through protein-protein interaction by STRING analysis. The complete list of all GO molecular functions, GO cellular components, and GO biological processes are visualised in Fig. S3. The immature biofilm mainly presented upregulation in binding functions and catalytic activity. Furthermore, biological processes primarily involved in metabolic processes, implicating a change in metabolism during biofilm growth. These results align with a previous study focused on the difference in proteome modulation during the growth stages of biofilm development [20]. The mature biofilm expressed an upregulation in toxin activity, which is believed to play a crucial role in biofilm antibiotic tolerance and resistance by disrupting cellular processes [46].

The tricarboxylic acid (TCA) cycle complex presented an upregulation in the mature biofilm. The TCA cycle is linked to the production of PIA synthesis, a critical extracellular matrix component for staphylococci. It is demonstrated that repressing the TCA cycle is linked to increased synthesis of PIA. Previously, the upregulation in the TCA cycle was linked to a decrease in PIA synthesis in *S. aureus* biofilms [20,47].

**Fig. 6:**
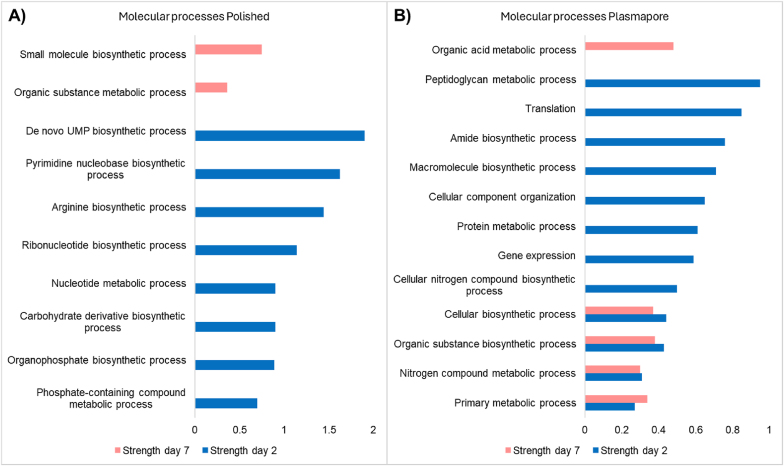
**Molecular and cellular functions upregulated per biofilm growth state grown on Polished and plasmapore discs.** GO molecular and cellular functions are upregulated in the mature biofilm and are shown in pink. Blue shows GO molecular and cellular functions upregulated in the immature biofilm.

Next, proteome changes between the mature and immature biofilms per surface type were investigated. Table S2 lists the differentially abundant proteins observed between different discs across days. GO molecular functions for the upregulated proteins in immature and mature biofilm grown on polished and plasmapore-coated material are visualised in Fig. 6, and a complete list of all GO molecular functions is represented in Fig. S4. Even though the biomass and bacterial count increased between the immature and mature biofilm grown on corundum discs, no change in GO processes was found based on proteome changes.

The immature biofilm, grown on polished Ti material, showed the upregulation of proteins involved in multiple molecular processes involved in pyrimidine synthesis, including de novo uridine monophosphate (UMP), pyrimidine nucleobase biosynthesis, and ribonucleotide biosynthetic process (Fig. 6A). Pyrimidines are aromatic compounds that constitute essential building blocks for DNA and RNA. It is suggested that the pyrimidine de novo synthesis plays a vital role in bacterial adherence and biofilm formation [[48], [49], [50]].

Furthermore, the biofilm grown on plasmapore-coated materials demonstrated an upregulation of 20 molecular processes, with the highest strength for the Peptidoglycan metabolic process and translation (Fig. 6B). The proteins upregulated in mature biofilm revealed involvement in nine molecular processes, with the highest strength for the Organic acid metabolic process, small molecule metabolic process, and Organic substance biosynthetic process.

### 3.4 Protein differences between discs

Finally, changes in protein abundance between biofilms grown on different surfaces were compared. This analysis was performed independently within the two growth stages, immature and mature biofilm. A multivariate analysis was performed, followed by hierarchical clustering.

Differentially abundant proteins were mainly observed in the immature biofilm, suggesting that protein changes primarily occur during the initial stage of biofilm formation. One protein, uncharacterised leukocidin-like protein 2, belonging to the Aerolysin family, was differentially expressed in mature biofilm between disc-type polished and plasmapore coated. Aerolysin is a group of bacterial proteins that can kill target cells [51]. Interestingly, the absence of modulated proteins suggests that the surface may have a limited influence on the biofilm proteome of more mature biofilms. The metabolic change between the maturation stage of the biofilm appears to account for the higher protein variability observed in the immature biofilm.

**Fig. 7:**
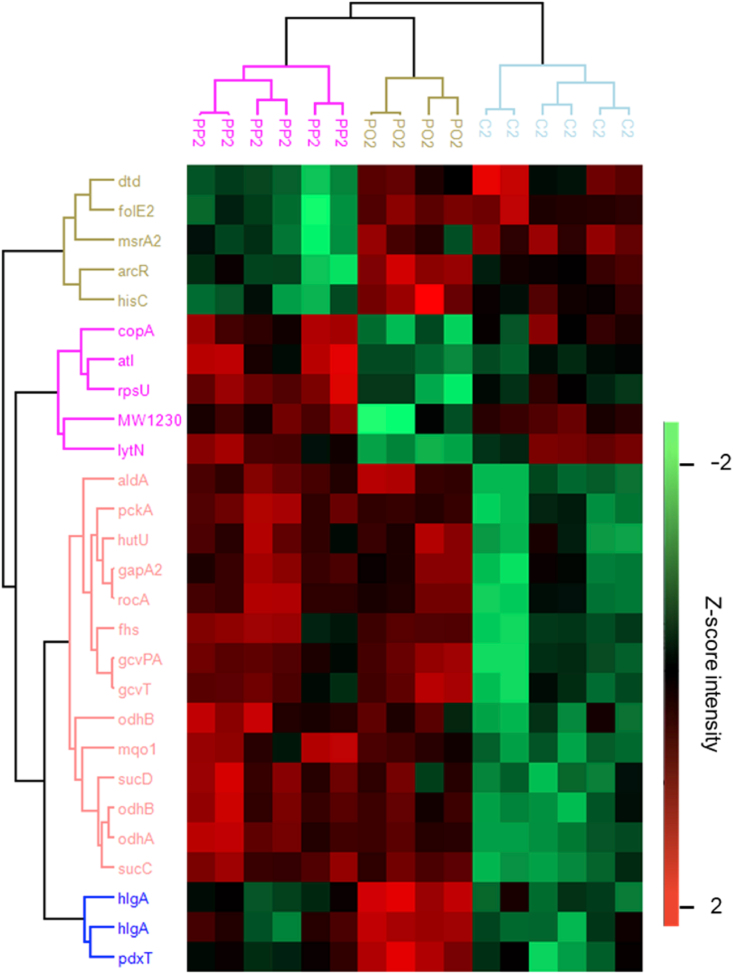
**Heatmap of modulated proteins between disctypes and corresponding enrichment analysis at 2 days.** The heatmaps visualizes the modulated proteins between the biofilms grown on different surfaces. The disc types Plasmapore-coated (PP2), polished (PO2), and corundum blasted (C2) are separated by the Euclidean distance column clustering. Furthermore, the row clustering separated by the Euclidean distance shows up (red) and down (green) regulation of protein clusters per disc roughness type.

The immature biofilm, grown for 2 days, demonstrated 27 deregulated proteins visualised in a heatmap in Fig. 7 and listed in Table 3S. This table describes the protein, cellular components, and molecules found in the literature that represent potential inhibition of this protein.

Fig. 7 highlights a prominent row-cluster of 14 proteins downregulated in biofilms formed on corundum materials compared to those on plasmapore-coated and polished surfaces. This finding suggests that corundum surfaces have the most pronounced effect on the biofilm's proteome in the initial stage of biofilm formation. Among the proteins analysed, four proteins that could be linked to the TCA cycle were found to be upregulated, Succinate-CoA ligase subunits alpha and beta (sucC and sucD), Dihydrolipoyllysine-residue succinyltransferase component of 2-oxoglutarate dehydrogenase complex (odhB) and 2-oxoglutarate dehydrogenase E1 component (odhA). Resch et al. described the upregulation of these genes in biofilm compared to planktonic cells [52,53]. SucC-mutated S. aureus biofilms had an increased amount of dead cells, indicating the importance of SucC in biofilm maintenance [54]. Additionally, the TCA cycle is particularly noteworthy because it suppresses the production of PIA, a critical component of the biofilm matrix.

Furthermore, bifunctional autolysis protein (Atl) and Probable cell wall hydrolase (LytN) appeared to be upregulated in the second-row cluster, which followed the same pattern as the biofilm growth. Here, bacteria exhibited the lowest CFU count on the polished material, increasing growth on the corundum blasted material and the highest growth on the plasmapore-coated material. Therefore, the relationship between these upregulated proteins and biofilm growth was demonstrated. Atl is a well-known surface protein of *S. aureus* and is known for its importance in the initial attachment [55,56]. This peptidoglycan is involved in cell wall degradation and separation during cell division and plays a vital role in the development of biofilms [57]. Given its critical role in the initial attachment of bacteria to abiotic surfaces, Atl emerges as an interesting candidate for the development of novel antimicrobial coatings. In literature, a novel antibacterial, diarulurea ZJ-2, was described. This antimicrobial is a peptidoglycan (PG) hydrolase, affecting atl-mediated PG homeostasis. Previously, Yang et al. demonstrated that ZJ-2 down-regulated the expression of peptidoglycan hydrolase through, among others, Atl [58,59]. Furthermore, the deregulated probable cell wall hydrolase LytN, which was reported to influence the bacterial cell wall. The disruption of LytN showed a decrease in cell growth and resulted in abnormalities in the cell wall. Recently, the involvement of LytN in the antibacterial mechanism of magnolol was described. Wang et al. have shown that biofilms following treatment with the antimicrobial agent magnolol decreased levels of LytN [60,61].

While the above-described compounds show promising results in the laboratory, translating these therapies to the clinical setting presents multiple challenges. A critical point in developing new antimicrobial coatings concerns the application and stabilisation of the coating [62]. Strategies for coating the implant surface include polymer brush coating, surface grafting, hydrogels and more. Another bottleneck in developing surface coatings is the coating failure in clinical conditions. Furthermore, the coatings can only release the antimicrobial agent for a limited period and will only deliver a bactericidal concentration for several days. Therefore, the coatings will only prevent early post-surgical infections [63]. Hence, future research should extend to coating properties and in vivo release studies. Furthermore, surface coatings for orthopaedic implants with nanomaterials hold great promise for the antimicrobial properties of Ti implants [[64], [65], [66]].

This study has certain limitations, including focusing on only two time points in biofilm development. Since the main changes were observed in the immature stage of the biofilm, future studies could explore additional time points in the immature biofilm to provide a more detailed understanding of biofilm formation in its early stages. Since metabolism changes significantly between time points, metabolomics could be valuable for gaining deeper insights into these metabolic processes. However, specific time points for biofilm development are not clearly defined or standardised in the current literature for biofilm systems. Therefore, for a valuable extension of this work with more meaningful time points, standardised temporal markers need to be established in the field. Additionally, the study examined only one ATCC *S. aureus* strain, which, while commonly used in comparative research, may not capture the full diversity seen in clinical strains. ATCC strains are standardised and well-characterised, allowing for reproducible and meaningful comparisons. However, investigating clinical strains could offer a broader perspective on biofilm development and variability.

## 4 Conclusion

MS-based proteomic analysis provided insight into the mechanistic changes between mature and immature biofilm grown on Ti substrates with various surface roughnesses commonly used for orthopaedic implants. Here, a proteomics workflow was optimised for *S. aureus* protein isolation and digestion, revealing that combining DDM with an automated SP3 protocol yielded the most favourable results.

This optimised proteomics workflow was employed to dive into the proteome changes of *S. aureus* infections. The 7-day mature biofilm demonstrated an overall increase in proteins linked to toxin activity and the TCA cycle. In contrast, the 2-day immature biofilm reflected increased binding and catalytic activities and metabolic processes, indicating a change in the bacterial metabolism during biofilm development.

Our findings demonstrate that the initial stage of biofilm formation is more responsive to Ti surface topography, with significant upregulation of proteins involved in adhesion and biofilm development on the corundum and plasmapore-coated surfaces. Potential target proteins can be found to inhibit biomolecular processes during the initial state of biofilm formation. In this initial stage, crucial proteins showed deregulation when the immature, two-day-old biofilms grown on the three surfaces were compared. This included multiple proteins already targeted in the literature for novel antimicrobial therapies, including Atl and lytN. These novel therapies can be tested as antimicrobial coatings for Ti surfaces in future research, resulting in targeted therapies per surface type.

The developed proteomics platform offers a powerful tool for future research to monitor bacterial and host cell responses to these coatings, advancing the development of innovative solutions for implant-associated infections. The proteomics platform can investigate the safety and efficiency of newly developed antimicrobial techniques.

## CRediT authorship contribution statement

**C. Kuik:** Writing – review & editing, Writing – original draft, Visualization, Methodology, Formal analysis, Conceptualization. **C. de Boer:** Visualization, Methodology, Formal analysis. **S.W.G. van Hoogstraten:** Writing – original draft, Methodology, Formal analysis. **K. Freulings:** Methodology, Data curation. **M. Honing:** Writing – review & editing, Supervision. **J.J.C. Arts:** Writing – review & editing, Project administration, Funding acquisition, Conceptualization. **B. Cillero-Pastor:** Writing – review & editing, Supervision, Funding acquisition, Conceptualization.

## Conflicts of interest

The authors declare no financial, commercial, or personal relationships that could be perceived as potential conflicts of interest related to the study ‘Proteomic Signatures of *Staphylococcus aureus* Biofilm Maturation on Orthopaedic Implants’.

## Data Availability

Data will be made available on request.

## References

[1] Long M., Rack H.J. (1998). Titanium alloys in total joint replacement—a materialsscience perspective. Biomaterials.

[2] Damiati L., Eales M.G., Nobbs A.H. (2018). Impact of surface topography and coating on osteogenesis and bacterial attachment on titanium implants. J Tissue Eng.

[3] Yoshinari M., Oda Y., Kato T. (2001). Influence of surface modifications to titanium on antibacterial activity in vitro. Biomaterials.

[4] Rabih O., Darouiche M.D. (2004). Treatment of infections associated with surgical implants. N Engl J Med.

[5] Peyyala R., Ebersole J.L. (2013). Multispecies biofilms and host responses: "discriminating the trees from the forest". Cytokine.

[6] Jamal M., Ahmad W., Andleeb S. (2018). Bacterial biofilm and associated infections. J Chin Med Assoc.

[7] Arciola C.R., Campoccia D., Montanaro L. (2018). Implant infections: adhesion, biofilm formation and immune evasion. Nat Rev Microbiol.

[8] Khoury A.E., Lam K., Ellis B. (1992). Prevention and control of bacterial infections associated with medical devices. ASAIO J.

[9] Staats A., Li D., Sullivan A.C. (2021). Biofilm formation in periprosthetic joint infections. Ann Jt.

[10] Kherabi Y., Zeller V., Kerroumi Y. (2022). Streptococcal and Staphylococcus aureus prosthetic joint infections: are they really different?. BMC Infect Dis.

[11] Triffault-Fillit C., Ferry T., Laurent F. (2019). Microbiologic epidemiology depending on time to occurrence of prosthetic joint infection: a prospective cohort study. Clin Microbiol Infect.

[12] Fadzil AFbA., Pramanik A., Basak A.K. (2022). Role of surface quality on biocompatibility of implants - a review. Ann 3D Print Med.

[13] Zhu X., Chen J., Scheideler L. (2004). Cellular reactions of osteoblasts to micron- and submicron-scale porous structures of titanium surfaces. Cells Tissues Organs.

[14] Wennerberg A., Albrektsson T. (2009). Effects of titanium surface topography on bone integration: a systematic review. Clin Oral Implants Res.

[15] Cooper L.F. (2000). A role for surface topography in creating and maintaining bone at titanium endosseous implants. J Prosthet Dent.

[16] van Hoogstraten S.W.G., Fechter J., Bargon R. (2024). The antibacterial properties of a silver multilayer coating for the prevention of bacterial biofilm formation on orthopedic implants—an in vitro study. Coatings (Oakv).

[17] Schubert O.T., Rost H.L., Collins B.C. (2017). Quantitative proteomics: challenges and opportunities in basic and applied research. Nat Protoc.

[18] Meissner F., Geddes-McAlister J., Mann M. (2022). The emerging role of mass spectrometry-based proteomics in drug discovery. Nat Rev Drug Discov.

[19] Rani A., Babu S. (2018). Environmental proteomic studies: closer step to understand bacterial biofilms. World J Microbiol Biotechnol.

[20] Rahman M.A., Amirkhani A., Chowdhury D. (2022). Proteome of Staphylococcus aureus biofilm changes significantly with aging. Int J Mol Sci.

[21] Broadbent J.A., Broszczak D.A., Tennakoon I.U. (2016). Pan-proteomics, a concept for unifying quantitative proteome measurements when comparing closely-related bacterial strains. Expert Rev Proteomics.

[22] Tsakou F., Jersie-Christensen R., Jenssen H. (2020). The role of proteomics in bacterial response to antibiotics. Pharmaceuticals.

[23] Semanjski M., Gratani F.L., Englert T. (2021). Proteome dynamics during antibiotic persistence and resuscitation. mSystems.

[24] Tsai C.F., Zhang P., Scholten D. (2021). Surfactant-assisted one-pot sample preparation for label-free single-cell proteomics. Commun Biol.

[25] Mosen P.R., Hardt R., Winter D. (2021). RapiGest precipitation depends on peptide concentration. Proteomics.

[26] Hughes C.S., Foehr S., Garfield D.A. (2014). Ultrasensitive proteome analysis using paramagnetic bead technology. Mol Syst Biol.

[27] Hughes C.S., Moggridge S., Muller T. (2019). Single-pot, solid-phase-enhanced sample preparation for proteomics experiments. Nat Protoc.

[28] Blankenburg S., Hentschker C., Nagel A. (2019). Improving proteome coverage for small sample amounts: an advanced method for proteomics approaches with low bacterial cell numbers. Proteomics.

[29] Abele M., Doll E., Bayer F.P. (2023). Unified workflow for the Rapid and in-depth characterization of bacterial proteomes. Mol Cell Proteomics.

[30] Hayoun K., Gouveia D., Grenga L. (2019). Evaluation of sample preparation methods for fast proteotyping of microorganisms by tandem mass spectrometry. Front Microbiol.

[31] Pino L.K., Just S.C., MacCoss M.J. (2020). Acquiring and analyzing data independent acquisition proteomics experiments without spectrum libraries. Mol Cell Proteomics.

[32] Demichev V., Messner C.B., Vernardis S.I. (2020). DIA-NN: neural networks and interference correction enable deep proteome coverage in high throughput. Nat Methods.

[33] Tyanova S., Temu T., Sinitcyn P. (2016). The Perseus computational platform for comprehensive analysis of (prote)omics data. Nat Methods.

[34] Niven D.J., McCormick T.J., Straus S.E. (2018). Reproducibility of clinical research in critical care: a scoping review. BMC Med.

[35] Poulos R.C., Hains P.G., Shah R. (2020). Strategies to enable large-scale proteomics for reproducible research. Nat Commun.

[36] Muller T., Kalxdorf M., Longuespee R. (2020). Automated sample preparation with SP3 for low-input clinical proteomics. Mol Syst Biol.

[37] Leid J.G., Shirtliff M.E., Costerton J.W. (2002). Human leukocytes adhere to, penetrate, and respond to Staphylococcus aureus biofilms. Infect Immun.

[38] Chen X., Thomsen T.R., Winkler H. (2020). Influence of biofilm growth age, media, antibiotic concentration and exposure time on Staphylococcus aureus and Pseudomonas aeruginosa biofilm removal in vitro. BMC Microbiol.

[39] Higashihira S., Simpson S.J., Collier C.D. (2022). Halicin is effective against Staphylococcus aureus biofilms in vitro. Clin Orthop Relat Res.

[40] Kim W.J., Kim S.H., Kang D.H. (2020). Thermal and non-thermal treatment effects on Staphylococcus aureus biofilms formed at different temperatures and maturation periods. Food Res Int.

[41] Otto M. (2018). Staphylococcal biofilms. Microbiol Spectr.

[42] Clarke S.R., Harris L.G., Richards R.G. (2002). Analysis of Ebh, a 1.1-megadalton cell wall-associated fibronectin-binding protein of Staphylococcus aureus. Infect Immun.

[43] Zhu Y., Weiss E.C., Otto M. (2007). Staphylococcus aureus biofilm metabolism and the influence of arginine on polysaccharide intercellular adhesin synthesis, biofilm formation, and pathogenesis. Infect Immun.

[44] Periasamy S., Chatterjee S.S., Cheung G.Y. (2012). Phenol-soluble modulins in staphylococci: what are they originally for?. Commun Integr Biol.

[45] Turner A.B., Gerner E., Firdaus R. (2022). Role of sodium salicylate in Staphylococcus aureus quorum sensing, virulence, biofilm formation and antimicrobial susceptibility. Front Microbiol.

[46] Qi X., Brothers K.M., Ma D. (2021). The Staphylococcus aureus toxin-antitoxin system YefM-YoeB is associated with antibiotic tolerance and extracellular dependent biofilm formation. J Bone Jt Infect.

[47] Zhu Y., Xiong Y.Q., Sadykov M.R. (2009). Tricarboxylic acid cycle-dependent attenuation of Staphylococcus aureus in vivo virulence by selective inhibition of amino acid transport. Infect Immun.

[48] Liu Y., Su S., Yu M. (2022). Pyrancoumarin derivative LP4C targeting of pyrimidine de novo synthesis pathway inhibits MRSA biofilm and virulence. Front Pharmacol.

[49] Yang H.J., Bogomolnaya L., McClelland M. (2017). De novo pyrimidine synthesis is necessary for intestinal colonization of Salmonella Typhimurium in chicks. PLoS One.

[50] Garavaglia M., Rossi E., Landini P. (2012). The pyrimidine nucleotide biosynthetic pathway modulates production of biofilm determinants in Escherichia coli. PLoS One.

[51] Parker M.W., van der Goot F.G., Buckley J.T. (1996). Aerolysin--the ins and outs of a model channel-forming toxin. Mol Microbiol.

[52] Resch A., Rosenstein R., Nerz C. (2005). Differential gene expression profiling of Staphylococcus aureus cultivated under biofilm and planktonic conditions. Appl Environ Microbiol.

[53] Campbell C., Fingleton C., Zeden M.S. (2021). Accumulation of succinyl coenzyme A perturbs the methicillin-resistant Staphylococcus aureus (MRSA) succinylome and is associated with increased susceptibility to beta-lactam antibiotics. mBio.

[54] De Backer S., Sabirova J., De Pauw I. (2018). Enzymes catalyzing the TCA- and urea cycle influence the matrix composition of biofilms formed by methicillin-resistant Staphylococcus aureus USA300. Microorganisms.

[55] Speziale P., Pietrocola G., Foster T.J. (2014). Protein-based biofilm matrices in Staphylococci. Front Cell Infect Microbiol.

[56] Houston P., Rowe S.E., Pozzi C. (2011). Essential role for the major autolysin in the fibronectin-binding protein-mediated Staphylococcus aureus biofilm phenotype. Infect Immun.

[57] Parvin F., Rahman M.A., Deva A.K. (2023). Staphylococcus aureus cell wall phenotypic changes associated with biofilm maturation and water availability: a key contributing factor for chlorine resistance. Int J Mol Sci.

[58] Xie Y., Wang L., Yang Y. (2022). Antibacterial and anti-biofilm activity of diarylureas against Enterococcus faecium by suppressing the gene expression of peptidoglycan hydrolases and adherence. Front Microbiol.

[59] Yang Y., Yao Z., Zhang J. (2024). Inhibiting peptidoglycan hydrolase alleviates MRSA pneumonia through autolysin-mediated MDP-NOD2 pathway. Infect Drug Resist.

[60] Wang D., Jin Q., Xiang H. (2011). Transcriptional and functional analysis of the effects of magnolol: inhibition of autolysis and biofilms in Staphylococcus aureus. PLoS One.

[61] Frankel M.B., Hendrickx A.P., Missiakas D.M. (2011). LytN, a murein hydrolase in the cross-wall compartment of Staphylococcus aureus, is involved in proper bacterial growth and envelope assembly. J Biol Chem.

[62] Campoccia D., Montanaro L., Arciola C.R. (2013). A review of the biomaterials technologies for infection-resistant surfaces. Biomaterials.

[63] Zhao L., Chu P.K., Zhang Y. (2009). Antibacterial coatings on titanium implants. J Biomed Mater Res B Appl Biomater.

[64] Kumar S.A.S., Praveenkumar K., Jothipandian S. (2025). Nanoscale surface modifications on Titanium plates- A strategy to mitigate MRSA biofilm-mediated implant infections: a pilot study. Microb Pathog.

[65] Sui J., Hou Y., Chen M. (2024). Nanomaterials for anti-infection in orthopedic implants: a review. Coatings (Oakv).

[66] Zhang L., Jin Z. (2024). Antibacterial activities of titanium dioxide (TiO(2)) nanotube with planar titanium silver (TiAg) to prevent orthopedic implant infection. J Orthop Surg Res.

