# Supplementary material for: Proteomic signatures of Staphylococcus aureus biofilm maturation on orthopaedic implants
Source: Biofilm. 2025 May 27;9:100287. doi: 10.1016/j.bioflm.2025.100287 (PMC12167111; doi:10.1016/j.bioflm.2025.100287)
Supplement: Multimedia component 1 [file mmc1.docx]

**Supplementary information**

**
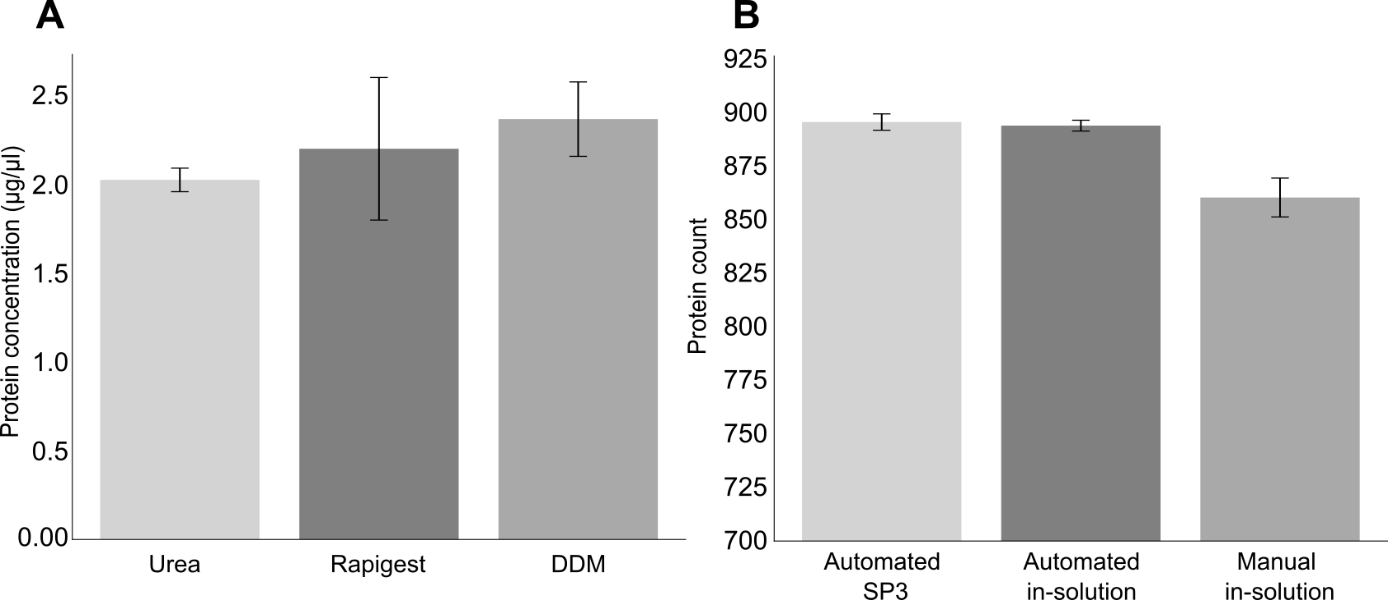
**

**Figure S1**: Protein recovery and protein count of bacterial cells. A) Protein concentration after cell lysis of biofilm bacterial cells using urea, urea with Rapigest or urea and DDM. B) Protein counts of biofilm bacteria after manual in solution.

**
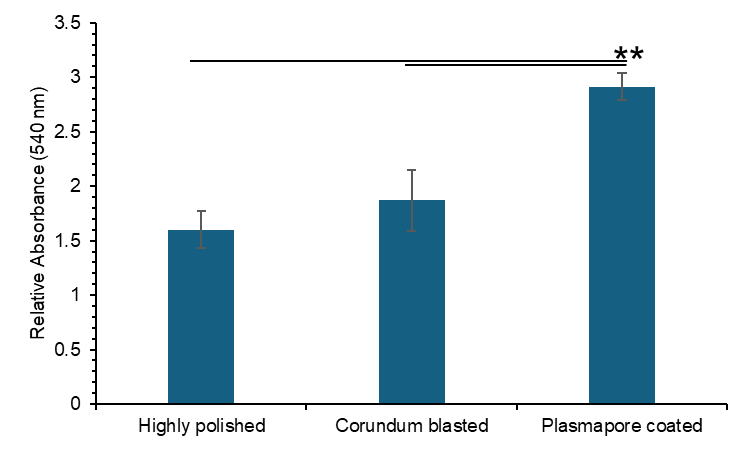
**

**Figure S2: Comparison of *S. aureus* ATCC 25923 biomass at 2-days of culture.** Bars represent the standard deviation of four replicates (** p < 0.005).

**

**Table S1 : Deregulated proteins between day 2 and 7 biofilm.** Including protein ID, protein description, gene name. t-test q value, student t-test difference, and the group in wich the protein is upregulated.

| Protein ID | Protein Description | Gene name | q-value | Student'sT-test Difference | Up in day |
| --- | --- | --- | --- | --- | --- |
| Q6G9U4 | Translation initiation factor IF-2 | infB | 0.038313 | 2.42097 | 2 |
| A6QG68 | Ornithine carbamoyltransferase | argF | 2.29E-08 | 1.87524 | 2 |
| Q2FYJ6 | Extracellular matrix-binding protein ebh | ebh | 1.02E-05 | 1.82244 | 2 |
| Q6GGX3 | Extracellular matrix-binding protein ebh | ebh | 2.66E-08 | 1.76128 | 2 |
| A5ISW6 | Extracellular matrix-binding protein ebh | ebh | 1.04E-07 | 1.75375 | 2 |
| A5ISW6 | Extracellular matrix-binding protein ebh | ebh | 4.16E-08 | 1.69499 | 2 |
| Q6GGX3 | Extracellular matrix-binding protein ebh | ebh | 2.99E-08 | 1.66203 | 2 |
| A6U1G3 | UPF0154 protein SaurJH1_1431 | MW1230 | 3.99E-07 | 1.61275 | 2 |
| A5ISW6 | Extracellular matrix-binding protein ebh | ebh | 2.99E-08 | 1.59617 | 2 |
| Q6GE63 | Uncharacterized lipoprotein SAR2457 | SAR2457 | 8.01E-16 | 1.58226 | 2 |
| Q2FXA4 | Coproporphyrin III ferrochelatase | cpfC | 0.000867 | 1.49876 | 2 |
| A5IUP4 | 3-hydroxyacyl-[acyl-carrier-protein] dehydratase FabZ | fabZ | 2.19E-06 | 1.43623 | 2 |
| A6QG68 | Ornithine carbamoyltransferase | arcB | 1.62E-05 | 1.38848 | 2 |
| Q2G131 | Efem/EfeO family lipoprotein | MW0319 | 1.03E-08 | 1.35447 | 2 |
| Q53630 | Elastin-binding protein EbpS | ebpS | 5.76E-05 | 1.33533 | 2 |
| A6QI23 | Foldase protein PrsA | prsA | 6.51E-07 | 1.33308 | 2 |
| A5ISW6 | Extracellular matrix-binding protein ebh | ebh | 5.98E-09 | 1.26857 | 2 |
| Q6GFL5 | Foldase protein PrsA | prsA | 6.40E-07 | 1.25548 | 2 |
| P38507 | Immunoglobulin G-binding protein A | spa | 0.001254 | 1.21096 | 2 |
| Q6GES1 | PTS system mannitol-specific EIICB component | mtlA | 5.84E-06 | 1.19008 | 2 |
| Q6GEA0 | Lysostaphin resistance protein A | lyrA | 7.90E-06 | 1.15443 | 2 |
| Q2FYJ6 | Extracellular matrix-binding protein ebh | ebh | 1.02E-05 | 1.11500 | 2 |
| A5ISE3 | Ribosome-recycling factor | frr | 0.000681 | 1.11069 | 2 |
| P60074 | UPF0291 protein SAV1341 | MW1228 | 5.72E-05 | 1.10328 | 2 |
| Q6GDG8 | Ornithine carbamoyltransferase, catabolic | arcB | 0.000131 | 1.09872 | 2 |
| Q6GE17 | 2,3-bisphosphoglycerate-dependent phosphoglycerate mutase | gpmA | 0.003287 | 1.08930 | 2 |
| A5INQ6 | Large ribosomal subunit protein bL9 | rplI | 7.48E-05 | 1.08425 | 2 |
| A6QGG8 | Translation initiation factor IF-2 | infB | 5.85E-06 | 1.00600 | 2 |
| Q2FV99 | Sortase A | srtA | 2.58E-09 | 0.974492 | 2 |
| Q5HFV0 | DNA-binding protein HU | hup | 3.35E-05 | 0.961948 | 2 |
| P11162 | PTS system lactose-specific EIICB component | lacE | 0.000269 | 0.946627 | 2 |
| A6QJK1 | Putative hemin import ATP-binding protein HrtA | hrtA | 0.001389 | 0.94398 | 2 |
| Q6G6F1 | D-lactate dehydrogenase | ldhD | 0.005952 | 0.94129 | 2 |
| Q6GHI8 | Probable cell wall hydrolase LytN | lytN | 0.007222 | 0.93169 | 2 |
| Q6GI62 | Serine protease HtrA-like | SAR0992 | 5.23E-08 | 0.930323 | 2 |
| Q2G1B9 | Ribulose-5-phosphate reductase 1 | tarJ | 0.000209 | 0.925684 | 2 |
| Q6GDK4 | 3-methyl-2-oxobutanoate hydroxymethyltransferase | panB | 7.92E-05 | 0.908263 | 2 |
| Q6GDP1 | Copper-exporting P-type ATPase | copA | 0.000764 | 0.907362 | 2 |
| Q2FV54 | O-acetyltransferase OatA | SACOL0978 | 0.002613 | 0.90645 | 2 |
| A6QH29 | Elastin-binding protein EbpS | ebpS | 1.34E-05 | 0.903411 | 2 |
| A5IUN6 | Membrane protein insertase YidC | yidC | 2.29E-08 | 0.898958 | 2 |
| A5IVG9 | Probable nitrate transporter NarT | narT | 6.19E-06 | 0.890633 | 2 |
| A5IQE1 | Coproheme decarboxylase | chdC | 5.86E-06 | 0.88527 | 2 |
| A5IQX2 | ATP-dependent Clp protease proteolytic subunit | clpP | 0.000155 | 0.880686 | 2 |
| Q2YTE1 | Acetyl-coenzyme A carboxylase carboxyl transferase subunit alpha | accA | 1.62E-05 | 0.878906 | 2 |
| Q6GG32 | Probable GTP-binding protein EngB | engB | 1.04E-05 | 0.878203 | 2 |
| P67592 | Tryptophan--tRNA ligase | trpS | 1.35E-05 | 0.875297 | 2 |
| A5IS03 | Protoheme IX farnesyltransferase | ctaB | 6.49E-06 | 0.874537 | 2 |
| A5ITA8 | Chaperone protein DnaK | dnaK | 0.000164 | 0.868694 | 2 |
| Q6GJN2 | Uncharacterized lipoprotein SAR0439 | SAR0439 | 0.012499 | 0.860043 | 2 |
| A6QFH3 | Glucose-6-phosphate isomerase | pgi | 0.001178 | 0.854242 | 2 |
| Q6GI31 | Bifunctional autolysin | atl | 0.000122 | 0.850594 | 2 |
| P0C1R5 | Galactose-6-phosphate isomerase subunit LacA | lacA | 0.00173 | 0.847464 | 2 |
| Q2G278 | Probable autolysin LDP | SAOUHSC_00773 | 0.004349 | 0.845646 | 2 |
| Q5HDV8 | Large ribosomal subunit protein uL3 | rplC | 0.00702 | 0.844461 | 2 |
| A5IV75 | Urease accessory protein UreD | ureD | 0.00732 | 0.830529 | 2 |
| Q6GGC1 | Chaperone protein DnaJ | dnaJ | 4.17E-05 | 0.826595 | 2 |
| Q2FZL3 | Staphopain B | sspB | 3.74E-05 | 0.823606 | 2 |
| A5IV74 | Urease accessory protein UreG | ureG | 0.00023 | 0.805575 | 2 |
| Q6GDH0 | Carbamate kinase 2 | arcC2 | 8.18E-05 | 0.805469 | 2 |
| Q6GG25 | Translation initiation factor IF-3 | infC | 8.49E-05 | 0.792847 | 2 |
| P67477 | Fructose-bisphosphate aldolase | fba | 0.002232 | 0.790544 | 2 |
| Q2FV54 | O-acetyltransferase OatA | oatA | 2.66E-08 | 0.787639 | 2 |
| Q2FI19 | Probable quinol oxidase subunit 3 | qoxC | 2.59E-05 | 0.785962 | 2 |
| A5ISN6 | Glycerol-3-phosphate acyltransferase | plsY | 0.000157 | 0.77406 | 2 |
| Q2FK71 | N-acetylmuramic acid 6-phosphate etherase | murQ | 2.34E-06 | 0.773462 | 2 |
| P0A0A1 | Pyruvate dehydrogenase E1 component subunit beta | pdhB | 0.012422 | 0.763738 | 2 |
| A6QIC8 | Glutamyl-tRNA(Gln) amidotransferase subunit A | gatA | 0.000106 | 0.761203 | 2 |
| O32421 | Probable cell wall amidase LytH | lytH | 2.21E-07 | 0.756305 | 2 |
| Q6GEE4 | Urease subunit alpha | ureC | 0.000376 | 0.753888 | 2 |
| Q2FF06 | Putative aldehyde dehydrogenase | MW2046 | 7.68E-05 | 0.753856 | 2 |
| Q6GG09 | Pyruvate kinase | pyk | 3.23E-05 | 0.750877 | 2 |
| A8Z3Z7 | UDP-N-acetylglucosamine--N-acetylmuramyl-(pentapeptide) pyrophosphoryl-undecaprenol N-acetylglucosamine transferase | murG | 2.43E-05 | 0.741624 | 2 |
| Q2FZ95 | Cell division protein FtsL | ftsL | 0.004148 | 0.74119 | 2 |
| Q2YTD2 | Uncharacterized peptidase SAB1567 | SAB1567 | 0.000941 | 0.735124 | 2 |
| Q6GGE2 | Probable endonuclease 4 | nfo | 0.006565 | 0.730279 | 2 |
| A5IRZ2 | Divalent metal cation transporter MntH | mntH | 0.000171 | 0.724433 | 2 |
| A5ITM3 | Acetyl-coenzyme A carboxylase carboxyl transferase subunit alpha | accA | 1.96E-07 | 0.722652 | 2 |
| Q2FEI5 | Putative formate dehydrogenase SAUSA300_2258 | SACOL2301 | 0.043513 | 0.72247 | 2 |
| Q6GDS2 | D-lactate dehydrogenase | ldhD | 0.043368 | 0.71879 | 2 |
| A5IT03 | GTPase Der | der | 5.18E-05 | 0.708919 | 2 |
| P63870 | Cysteine synthase | cysK | 5.98E-09 | 0.703709 | 2 |
| A7WYS8 | Lysine--tRNA ligase | lysS | 5.90E-05 | 0.699371 | 2 |
| P63729 | Carbamoyl phosphate synthase small chain | carA | 0.000164 | 0.699171 | 2 |
| A5IUH7 | Co-chaperonin GroES | groES | 0.01417 | 0.69437 | 2 |
| Q6GJB4 | Probable branched-chain-amino-acid aminotransferase | ilvE | 5.72E-05 | 0.693302 | 2 |
| P0C0Z1 | Response regulator protein VraR | vraR | 0.019206 | 0.693115 | 2 |
| P64333 | Delta-aminolevulinic acid dehydratase | hemB | 1.42E-05 | 0.690812 | 2 |
| Q2FVY9 | HTH-type transcriptional regulator SarV | sarV | 1.90E-05 | 0.682303 | 2 |
| A5ISC3 | Small ribosomal subunit protein bS16 | rpsP | 0.003977 | 0.675964 | 2 |
| Q5HGH0 | Phosphatidate cytidylyltransferase | cdsA | 0.000683 | 0.675203 | 2 |
| P0A0K4 | Thioredoxin | trxA | 0.019567 | 0.672616 | 2 |
| Q6G8Q5 | Porphobilinogen deaminase | hemC | 0.000164 | 0.665487 | 2 |
| A8Z3Z7 | UDP-N-acetylglucosamine--N-acetylmuramyl-(pentapeptide) pyrophosphoryl-undecaprenol N-acetylglucosamine transferase | murG | 8.90E-07 | 0.665388 | 2 |
| Q2FVB8 | Undecaprenyl phosphate transporter A | uptA | 0.011919 | 0.661773 | 2 |
| P14503 | Uncharacterized 27.7 kDa protein | | 0.01385 | 0.6582 | 2 |
| Q6GGZ0 | UDP-N-acetylglucosamine--N-acetylmuramyl-(pentapeptide) pyrophosphoryl-undecaprenol N-acetylglucosamine transferase | murG | 0.000189 | 0.653276 | 2 |
| Q6GGY3 | Peptide methionine sulfoxide reductase MsrA 2 | msrA2 | 2.59E-05 | 0.647084 | 2 |
| A5IV30 | Small ribosomal subunit protein uS19 | rpsS | 0.00463 | 0.644095 | 2 |
| Q6G8L9 | Uncharacterized peptidase SAS1635 | MW1651 | 0.002751 | 0.641832 | 2 |
| P63332 | Putative zinc metalloprotease SAV1262 | MW1145 | 0.006133 | 0.637568 | 2 |
| Q6G9U4 | Translation initiation factor IF-2 | infB | 0.00198 | 0.635004 | 2 |
| A5IV16 | Large ribosomal subunit protein uL30 | rpmD | 0.004863 | 0.629977 | 2 |
| Q6GG85 | Alanine--tRNA ligase | alaS | 0.004054 | 0.628983 | 2 |
| Q6GHG4 | tRNA pseudouridine synthase B | truB | 0.000193 | 0.62844 | 2 |
| Q5HF12 | HTH-type transcriptional regulator rot | rot | 5.72E-05 | 0.623038 | 2 |
| P60875 | Uncharacterized protein SAV2627.1 | MW2548 | 0.00241 | 0.622582 | 2 |
| Q6GDQ0 | ATP-dependent Clp protease ATP-binding subunit ClpL | clpL | 3.38E-05 | 0.619269 | 2 |
| P0C281 | ATP-dependent Clp protease ATP-binding subunit ClpC | clpC | 0.001187 | 0.616659 | 2 |
| Q6GH64 | Transketolase | tkt | 0.001729 | 0.616345 | 2 |
| A5IP58 | PTS system glucose-specific EIICBA component | ptsG | 1.04E-07 | 0.613426 | 2 |
| Q6GFV3 | tRNA (guanine-N(7)-)-methyltransferase | trmB | 0.003136 | 0.612682 | 2 |
| A6QJ92 | Large ribosomal subunit protein uL3 | rplC | 0.006132 | 0.609701 | 2 |
| Q6GKU4 | Chromosomal replication initiator protein DnaA | dnaA | 0.000349 | 0.60108 | 2 |
| P64164 | DNA translocase FtsK | ftsK | 0.001775 | 0.598311 | 2 |
| A6QH17 | Chorismate synthase | aroC | 0.002856 | 0.596038 | 2 |
| P0A029 | Cell division protein FtsZ | ftsZ | 2.36E-06 | 0.592255 | 2 |
| A5IS88 | Carbamoyl phosphate synthase large chain | carB | 0.002444 | 0.591008 | 2 |
| Q2FXP9 | Translation initiation factor IF-3 | infC | 0.000146 | 0.583308 | 2 |
| Q6GHD4 | Aerobic glycerol-3-phosphate dehydrogenase | glpD | 1.04E-05 | 0.580016 | 2 |
| A9JX08 | Phenol-soluble modulin alpha 4 peptide | psmA4 | 3.07E-05 | -4.6035 | 7 |
| A8Z0V1 | Phenol-soluble modulin alpha 1 peptide | psmA1 | 0.000611 | -3.35611 | 7 |
| P0A0M1 | Delta-hemolysin | hld | 0.000341 | -3.24242 | 7 |
| P69775 | Protein map | map | 5.87E-06 | -3.08136 | 7 |
| P0A0I5 | Accessory gene regulator protein A | agrA | 2.80E-07 | -2.86642 | 7 |
| Q6GFB8 | Protein map | map | 9.64E-05 | -2.55897 | 7 |
| Q2FFH9 | Sodium-dependent dicarboxylate transporter SdcS | sdcS | 0.000854 | -2.43592 | 7 |
| P65288 | Lipase 1 | lip1 | 0.005889 | -2.32873 | 7 |
| P0A071 | Gamma-hemolysin component A | hlgA | 0.001048 | -2.17246 | 7 |
| Q6GE13 | Gamma-hemolysin component C | hlgC | 5.33E-06 | -2.10405 | 7 |
| Q2YV12 | PTS system MurNAc-GlcNAc-specific EIIBC component | SAB0132 | 0.000181 | -2.07604 | 7 |
| A0A0H2XHV5 | 6-phospho-N-acetylmuramidase | mupG | 3.11E-05 | -2.0413 | 7 |
| Q53599 | Protein map | map | 0.002024 | -1.85788 | 7 |
| Q6GE15 | Immunoglobulin-binding protein Sbi | sbi | 1.40E-06 | -1.79156 | 7 |
| P69775 | Protein map | map | 5.99E-05 | -1.76499 | 7 |
| P65288 | Lipase 1 | lip1 | 0.029088 | -1.75995 | 7 |
| Q5HES4 | Fumarate hydratase class II | fumC | 0.035518 | -1.73338 | 7 |
| Q6GJZ6 | Lipase 2 | lip2 | 0.018258 | -1.72459 | 7 |
| P0A071 | Gamma-hemolysin component A | hlgA | 0.000683 | -1.64127 | 7 |
| Q2YVZ4 | Immunoglobulin-binding protein Sbi | sbi | 3.38E-06 | -1.59546 | 7 |
| Q2YXE6 | Uncharacterized N-acetyltransferase SAB1040c | MW1059 | 1.31E-05 | -1.52891 | 7 |
| A5ITE0 | UPF0473 protein SaurJH9_1672 | MW1565 | 0.000435 | -1.52846 | 7 |
| A5IRZ9 | UPF0358 protein SaurJH9_1172 | MW0995 | 0.000225 | -1.47703 | 7 |
| A6QIN3 | Accessory gene regulator protein B | agrB | 0.011882 | -1.38269 | 7 |
| A5IRJ1 | Putative phosphoesterase SaurJH9_1013 | MW0896 | 9.64E-05 | -1.32287 | 7 |
| Q2YT09 | Endoribonuclease YbeY | ybeY | 2.20E-05 | -1.30404 | 7 |
| P09616 | Alpha-hemolysin | hly | 0.03784 | -1.29714 | 7 |
| A5ITD0 | 5-oxoprolinase subunit A | pxpA | 9.77E-05 | -1.23479 | 7 |
| Q6GE66 | Probable malate:quinone oxidoreductase 1 | mqo1 | 2.12E-06 | -1.20472 | 7 |
| Q6GGH6 | Exodeoxyribonuclease 7 small subunit | xseB | 0.001328 | -1.19851 | 7 |
| Q6GFE8 | Staphopain A | sspP | 0.001015 | -1.19235 | 7 |
| Q6GE12 | Gamma-hemolysin component B | hlgB | 8.59E-05 | -1.1757 | 7 |
| A0A0H2XI99 | Type VII secretion system extracellular protein A | esxA | 0.040724 | -1.17187 | 7 |
| P0A071 | Gamma-hemolysin component A | hlgA | 0.000202 | -1.14698 | 7 |
| Q6GHJ9 | UPF0122 protein SAR1212 | SAR1212 | 6.40E-07 | -1.14667 | 7 |
| Q2FH01 | L-threonine dehydratase catabolic TdcB | tdcB | 0.000542 | -1.12933 | 7 |
| Q6GK24 | Type VII secretion system protein EssC | essC | 0.008173 | -1.09066 | 7 |
| Q2YUL2 | Thiamine-phosphate synthase | thiE | 4.69E-06 | -1.09029 | 7 |
| Q6GKC2 | N-acetyl-gamma-glutamyl-phosphate reductase | argC | 5.00E-05 | -1.06176 | 7 |
| Q6GFX3 | Acetoin utilization protein AcuC | acuC | 5.84E-06 | -1.05236 | 7 |
| Q6GII7 | 3-dehydroquinate dehydratase | aroD | 6.50E-09 | -1.04382 | 7 |
| A6QEK4 | Ribulokinase | araB | 1.96E-07 | -1.03451 | 7 |
| Q2YT09 | Endoribonuclease YbeY | ybeY | 0.000126 | -1.01415 | 7 |
| A5ISV2 | UPF0346 protein SaurJH9_1481 | MW1311 | 0.001389 | -1.0089 | 7 |
| A5ISD0 | Succinate--CoA ligase [ADP-forming] subunit beta | sucC | 9.88E-07 | -0.99826 | 7 |
| P64375 | Acetoin utilization protein AcuC | acuC | 0.000253 | -0.99123 | 7 |
| A5IQ73 | Pyridoxal 5'-phosphate synthase subunit PdxT | pdxT | 1.63E-06 | -0.98428 | 7 |
| A5IRB2 | D-alanyl carrier protein | dltC | 6.12E-05 | -0.96687 | 7 |
| Q2FH83 | Uncharacterized protein SAUSA300_1248 | MW1239 | 5.45E-05 | -0.94178 | 7 |
| Q6GKM5 | Uncharacterized chromosomal cassette SCCmec type IVc protein CR006 | CR006 | 0.000163 | -0.94005 | 7 |
| Q6GG12 | Isocitrate dehydrogenase [NADP] | icd | 2.97E-07 | -0.93963 | 7 |
| A5ISW6 | Extracellular matrix-binding protein ebh | ebh | 3.54E-05 | -0.93733 | 7 |
| A5IUS3 | Probable DNA-directed RNA polymerase subunit delta | rpoE | 0.004814 | -0.92317 | 7 |
| Q6GFK5 | Fumarate hydratase class II | fumC | 7.07E-06 | -0.91427 | 7 |
| Q6GHS9 | Fibrinogen-binding protein | fib | 0.000368 | -0.91081 | 7 |
| A6QED4 | Recombination protein RecR | recR | 0.000288 | -0.8986 | 7 |
| Q2G0I9 | Octanoyl-[GcvH]:protein N-octanoyltransferase | lipL | 4.50E-08 | -0.87925 | 7 |
| P64180 | Glyceraldehyde-3-phosphate dehydrogenase 2 | gapA2 | 2.00E-05 | -0.87376 | 7 |
| P09978 | Phospholipase C | hlb | 4.70E-05 | -0.86621 | 7 |
| A5IPD8 | D-ribose pyranase | rbsD | 0.00057 | -0.86281 | 7 |
| Q6GJM1 | Probable inorganic carbon transporter subunit DabA | dabA | 4.16E-08 | -0.85486 | 7 |
| A5IW58 | HTH-type transcriptional regulator ArcR | arcR | 0.003297 | -0.8499 | 7 |
| A6QGU6 | 4-hydroxy-tetrahydrodipicolinate synthase | dapA | 0.030773 | -0.84631 | 7 |
| A5IS36 | Glutamate racemase | murI | 0.000175 | -0.8454 | 7 |
| A5IUL7 | Holo-[acyl-carrier-protein] synthase | acpS | 3.27E-07 | -0.8359 | 7 |
| P60297 | Ornithine aminotransferase 2 | rocD2 | 0.005924 | -0.81801 | 7 |
| Q5HF07 | Riboflavin biosynthesis protein RibBA | ribBA | 0.000118 | -0.81482 | 7 |
| Q6GDH2 | Clumping factor B | clfB | 0.011919 | -0.79414 | 7 |
| A5ISS8 | 4-hydroxy-tetrahydrodipicolinate reductase | dapB | 0.019386 | -0.78821 | 7 |
| Q6GH32 | Tryptophan synthase alpha chain | trpA | 0.001884 | -0.78625 | 7 |
| A5IQR6 | 7-cyano-7-deazaguanine synthase | queC | 0.019493 | -0.78556 | 7 |
| P60297 | Ornithine aminotransferase 2 | rocD2 | 0.004946 | -0.77574 | 7 |
| Q6GGW9 | Alanine dehydrogenase 1 | ald1 | 7.90E-06 | -0.77065 | 7 |
| P0A0Q1 | Uncharacterized protein SAV1055 | MW0938 | 0.001502 | -0.769 | 7 |
| P60070 | Anti-sigma-B factor antagonist | rsbV | 0.005817 | -0.76663 | 7 |
| P0C000 | Response regulator ArlR | arlR | 2.01E-07 | -0.76305 | 7 |
| Q2FUW3 | Accessory Sec system protein Asp1 | asp1 | 7.68E-05 | -0.76003 | 7 |
| P56740 | Dihydroneopterin aldolase | folB | 1.29E-05 | -0.75934 | 7 |
| Q2G0P7 | Protein-arginine kinase activator protein | mcsA | 0.000111 | -0.7496 | 7 |
| A6QFL5 | UPF0738 protein NWMN_0875 | MW0886 | 7.70E-06 | -0.74078 | 7 |
| A6QHC5 | Heat-inducible transcription repressor HrcA | hrcA | 0.004946 | -0.73531 | 7 |
| Q5HGG8 | Proline--tRNA ligase | proS | 0.006158 | -0.73206 | 7 |
| A5IV21 | Small ribosomal subunit protein uS14B | rpsZ | 0.043522 | -0.72865 | 7 |
| Q2FH36 | Cold shock protein CspA | cspA | 0.027893 | -0.72856 | 7 |
| A5IVB2 | Imidazolonepropionase | hutI | 0.001031 | -0.72764 | 7 |
| Q2FIT4 | Response regulator SaeR | saeR | 0.00018 | -0.71712 | 7 |
| A5ITB0 | Heat-inducible transcription repressor HrcA | hrcA | 5.18E-08 | -0.71407 | 7 |
| Q2G2X2 | Glycerol-3-phosphate cytidylyltransferase | tarD | 2.10E-07 | -0.70967 | 7 |
| A6QGE1 | Large ribosomal subunit protein bL19 | rplS | 0.0065 | -0.70767 | 7 |
| Q2FYJ2 | Alanine dehydrogenase 1 | ald1 | 1.22E-05 | -0.6935 | 7 |
| A5IU69 | Sodium/proline symporter | putP | 0.017685 | -0.69154 | 7 |
| A7WXH5 | N-acetyl-gamma-glutamyl-phosphate reductase | argC | 0.00011 | -0.6866 | 7 |
| Q2FUW5 | Accessory Sec system protein Asp3 | asp3 | 0.000218 | -0.68175 | 7 |
| P65421 | Probable malate:quinone oxidoreductase 1 | mqo1 | 1.64E-06 | -0.67656 | 7 |
| A5IPT7 | 5-methyltetrahydropteroyltriglutamate--homocysteine methyltransferase | metE | 0.000792 | -0.67477 | 7 |
| Q6GBX4 | Dihydropteroate synthase | folP | 0.038791 | -0.67331 | 7 |
| A6U565 | ATP phosphoribosyltransferase regulatory subunit | hisZ | 6.98E-06 | -0.67162 | 7 |
| Q6GG04 | DNA polymerase III subunit alpha | dnaE | 2.19E-06 | -0.66119 | 7 |
| A6QJC0 | Sulfur carrier protein FdhD | fdhD | 0.000267 | -0.65922 | 7 |
| A5IS08 | UPF0298 protein SaurJH9_1181 | MW1004 | 0.001087 | -0.64883 | 7 |
| Q2YZ69 | Imidazole glycerol phosphate synthase subunit HisH | hisH | 0.001808 | -0.64826 | 7 |
| A5IQ98 | RNA-binding protein SaurJH9_0567 | MW0499 | 0.000169 | -0.64189 | 7 |
| A5IPU8 | Small ribosomal subunit protein bS18 | rpsR | 0.017171 | -0.64052 | 7 |
| A0A0H2WWB5 | o-succinylbenzoate synthase | menC | 2.97E-07 | -0.64029 | 7 |
| Q6G9I7 | Tryptophan synthase beta chain | trpB | 0.009004 | -0.63771 | 7 |
| A5ISV8 | Thymidylate synthase | thyA | 2.74E-06 | -0.63391 | 7 |
| A6QED4 | Recombination protein RecR | recR | 0.006241 | -0.6321 | 7 |
| A5ITW2 | 2-succinylbenzoate--CoA ligase | menE | 6.51E-07 | -0.62624 | 7 |
| A6QGW6 | 2-oxoglutarate dehydrogenase E1 component | odhA | 0.000362 | -0.62256 | 7 |
| P0A0P9 | Lactose phosphotransferase system repressor | lacR | 8.30E-06 | -0.61111 | 7 |
| Q6G625 | Protein translocase subunit SecA 2 | secA2 | 0.000209 | -0.60794 | 7 |
| Q6GGG3 | Probable glycine dehydrogenase (decarboxylating) subunit 1 | gcvPA | 2.34E-06 | -0.59851 | 7 |
| A5IQ06 | Probable inorganic carbon transporter subunit DabA | dabA | 1.91E-07 | -0.59462 | 7 |
| Q6GH70 | Small ribosomal subunit protein uS14A | rpsN | 0.040724 | -0.59335 | 7 |
| Q69HU0 | Probable copper-transporting P-type ATPase B | copB | 0.006233 | -0.58614 | 7 |
| Q5HES4 | Fumarate hydratase class II | fumC | 5.72E-05 | -0.58467 | 7 |
| Q2FIC1 | Putative peptidyl-prolyl cis-trans isomerase | MW0836 | 0.001341 | -0.58445 | 7 |
| Q2G2M4 | Mini-ribonuclease 3 | mrnC | 0.000157 | -0.58321 | 7 |
| Q6GE99 | Ribose-5-phosphate isomerase A | rpiA | 1.96E-07 | -0.58297 | 7 |
| A5IPX0 | GMP synthase [glutamine-hydrolyzing] | guaA | 9.88E-07 | -0.58105 | 7 |
| A5IW37 | Oxygen-dependent choline dehydrogenase | betA | 0.000126 | -0.58076 | 7 |

**
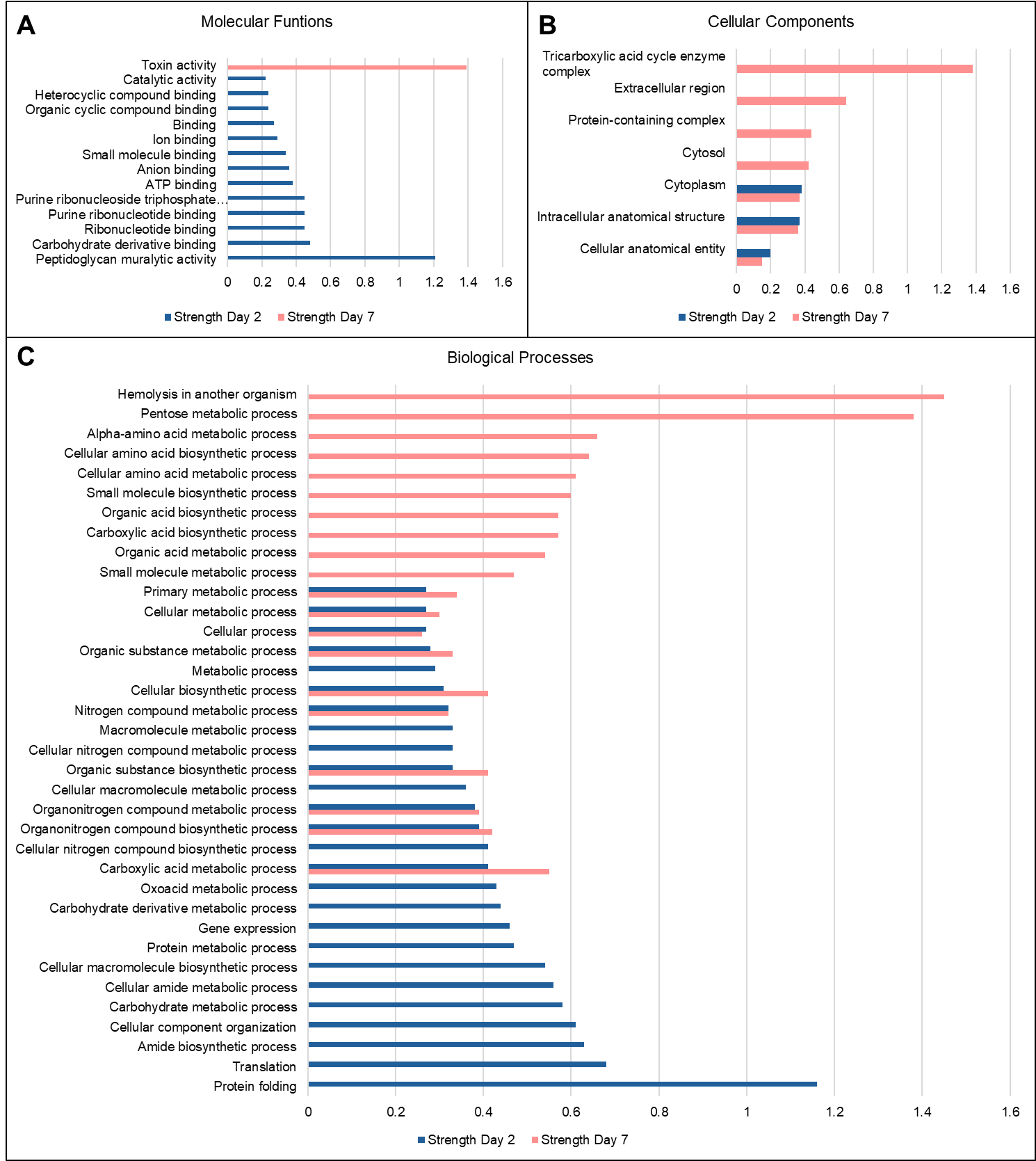
**

**Figure S3: Complete list of all GO terms.** A) GO molecular functions upregulated un Day 7 and day 2. B) The GO cellular components in which the deregulated proteins are located. C) Cellular processes in which the deregulated proteins are involved.

**Table S2 : differentially abundant proteins found between different disks between days.** Including protein ID, protein description, gene name. t-test q value, student t-test difference, and the group in wich the protein is upregulated.

| Disk type | Protein ID | Protein description | Gene name | Student's T-test q-value | Student's T-test Difference | Up in day |
| --- | --- | --- | --- | --- | --- | --- |
| PO | A5IQE1 | Coproheme decarboxylase | chdC | 0.0438325 | 1.10512 | 2 |
| PO | A5IS85 | Aspartate carbamoyltransferase catalytic subunit | pyrB | 0.0114383 | 1.02626 | 2 |
| PO | A5IS88 | Carbamoyl phosphate synthase large chain | carB | 0.0116692 | 1.10509 | 2 |
| PO | A5ISW6 | Extracellular matrix-binding protein ebh | ebh | 0.0213723 | 2.01833 | 2 |
| PO | A5ISW6 | Extracellular matrix-binding protein ebh | ebh | 0.0183455 | 1.85625 | 2 |
| PO | A5ISW6 | Extracellular matrix-binding protein ebh | ebh | 0.0178162 | 1.58655 | 2 |
| PO | A5ISW6 | Extracellular matrix-binding protein ebh | ebh | 0.0256709 | 2.09532 | 2 |
| PO | A5ITA8 | Chaperone protein DnaK | dnaK | 0.0223921 | 0.960032 | 2 |
| PO | A5IUN6 | Membrane protein insertase YidC | yidC | 0.0354232 | 0.87893 | 2 |
| PO | A5IUP4 | 3-hydroxyacyl-[acyl-carrier-protein] dehydratase FabZ | fabZ | 0.00865474 | 1.74147 | 2 |
| PO | A5IUP7 | ATP synthase epsilon chain | atpC | 0.0131656 | 0.825216 | 2 |
| PO | A5IUP9 | ATP synthase gamma chain | atpG | 0.0281679 | 0.890875 | 2 |
| PO | A5IV74 | Urease accessory protein UreG | ureG | 0.0345701 | 0.727074 | 2 |
| PO | A6QD96 | HTH-type transcriptional regulator SarS | sarS | 0.0435823 | 0.914826 | 2 |
| PO | A6QG68 | Ornithine carbamoyltransferase | arcB | 0.024596 | 1.79547 | 2 |
| PO | A6QG68 | Ornithine carbamoyltransferase | argF | 0.0200909 | 2.16014 | 2 |
| PO | A6QH29 | Elastin-binding protein EbpS | ebpS | 0.0496971 | 0.907818 | 2 |
| PO | A6QK27 | PTS system glucoside-specific EIICBA component | glcB | 0.0172087 | 0.747279 | 2 |
| PO | A6U1G3 | UPF0154 protein SaurJH1_1431 | MW1230 | 0.0438325 | 1.29917 | 2 |
| PO | A8Z3Z7 | UDP-N-acetylglucosamine--N-acetylmuramyl-(pentapeptide) pyrophosphoryl-undecaprenol N-acetylglucosamine transferase | murG | 0.024596 | 0.759504 | 2 |
| PO | A8Z3Z7 | UDP-N-acetylglucosamine--N-acetylmuramyl-(pentapeptide) pyrophosphoryl-undecaprenol N-acetylglucosamine transferase | murG | 0.0172087 | 0.721688 | 2 |
| PO | P0A039 | Glutamine synthetase | glnA | 0.0256709 | 0.940102 | 2 |
| PO | P0A0K4 | Thioredoxin | trxA | 0.0478699 | 1.09685 | 2 |
| PO | P11162 | PTS system lactose-specific EIICB component | lacE | 0.0435823 | 0.789595 | 2 |
| PO | P38507 | Immunoglobulin G-binding protein A | spa | 0.0461483 | 1.55892 | 2 |
| PO | P63729 | Carbamoyl phosphate synthase small chain | carA | 0.0193241 | 1.17001 | 2 |
| PO | P63870 | Cysteine synthase | cysK | 0.024596 | 0.697037 | 2 |
| PO | P65905 | Dihydroorotase | pyrC | 0.0105637 | 0.966695 | 2 |
| PO | P65916 | Orotate phosphoribosyltransferase | pyrE | 0.0435823 | 0.659095 | 2 |
| PO | Q2FV54 | O-acetyltransferase OatA | oatA | 0.0256709 | 0.674649 | 2 |
| PO | Q2FV99 | Sortase A | srtA | 0.00471752 | 0.905447 | 2 |
| PO | Q2FVY9 | HTH-type transcriptional regulator SarV | sarV | 0.00865474 | 0.959628 | 2 |
| PO | Q2G131 | Efem/EfeO family lipoprotein | MW0319 | 0.0330385 | 1.12227 | 2 |
| PO | Q2YXS9 | GMP reductase | guaC | 0.0339827 | 0.633838 | 2 |
| PO | Q6GDB7 | UPF0312 protein SAR2769 | SAR2769 | 0.0183455 | 0.633176 | 2 |
| PO | Q6GDG8 | Ornithine carbamoyltransferase, catabolic | arcB | 0.0435823 | 1.52142 | 2 |
| PO | Q6GDQ0 | ATP-dependent Clp protease ATP-binding subunit ClpL | clpL | 0.0416478 | 0.622689 | 2 |
| PO | Q6GE63 | Uncharacterized lipoprotein SAR2457 | SAR2457 | 0.0033399 | 1.71495 | 2 |
| PO | Q6GFV3 | tRNA (guanine-N(7)-)-methyltransferase | trmB | 0.0330385 | 0.764188 | 2 |
| PO | Q6GGX3 | Extracellular matrix-binding protein ebh | ebh | 0.0178162 | 1.92448 | 2 |
| PO | Q6GGX3 | Extracellular matrix-binding protein ebh | ebh | 0.0200909 | 2.10895 | 2 |
| PO | Q6GGY3 | Peptide methionine sulfoxide reductase MsrA 2 | msrA2 | 0.0366763 | 0.754362 | 2 |
| PO | Q6GGZ0 | UDP-N-acetylglucosamine--N-acetylmuramyl-(pentapeptide) pyrophosphoryl-undecaprenol N-acetylglucosamine transferase | murG | 0.0366763 | 0.679517 | 2 |
| PO | Q6GKJ3 | HTH-type transcriptional regulator SarS | sarS | 0.0363645 | 0.717928 | 2 |
| PO | A5IPD8 | D-ribose pyranase | rbsD | 0.0463875 | -0.882516 | 7 |
| PO | A5IQ06 | Probable inorganic carbon transporter subunit DabA | dabA | 0.0330385 | -0.583087 | 7 |
| PO | A5IQ73 | Pyridoxal 5'-phosphate synthase subunit PdxT | pdxT | 0.044947 | -0.755799 | 7 |
| PO | A5IRZ9 | UPF0358 protein SaurJH9_1172 | MW0995 | 0.0323332 | -1.38948 | 7 |
| PO | A5ISD0 | Succinate--CoA ligase [ADP-forming] subunit beta | sucC | 0.0222211 | -0.813212 | 7 |
| PO | A5ISV2 | UPF0346 protein SaurJH9_1481 | MW1311 | 0.0105637 | -1.01955 | 7 |
| PO | A5ITB0 | Heat-inducible transcription repressor HrcA | hrcA | 0.0363645 | -0.647647 | 7 |
| PO | A5ITE0 | UPF0473 protein SaurJH9_1672 |  | 0.0400763 | -1.57708 | 7 |
| PO | A5IUL7 | Holo-[acyl-carrier-protein] synthase | acpS | 0.00865474 | -0.928983 | 7 |
| PO | A6QED4 | Recombination protein RecR | recR | 0.0476793 | -0.621479 | 7 |
| PO | A6QED4 | Recombination protein RecR | recR | 0.0222211 | -0.824203 | 7 |
| PO | A6QEK4 | Ribulokinase | araB | 0.0178162 | -0.80272 | 7 |
| PO | A6QFL5 | UPF0738 protein NWMN_0875 | MW0886 | 0.0268557 | -0.586692 | 7 |
| PO | A6QHC5 | Heat-inducible transcription repressor HrcA | hrcA | 0.00920777 | -1.65147 | 7 |
| PO | A6QIN3 | Accessory gene regulator protein B | agrB | 0.0223921 | -2.28823 | 7 |
| PO | A6U565 | ATP phosphoribosyltransferase regulatory subunit | hisZ | 0.0498764 | -0.800789 | 7 |
| PO | A7WXH5 | N-acetyl-gamma-glutamyl-phosphate reductase | argC | 0.0230042 | -0.81484 | 7 |
| PO | A9JX08 | Phenol-soluble modulin alpha 4 peptide | psmA4 | 0.0316148 | -3.45377 | 7 |
| PO | P0A0I5 | Accessory gene regulator protein A | agrA | 0.0105637 | -2.49786 | 7 |
| PO | P0A0M1 | Delta-hemolysin | hld | 0.0416478 | -2.81234 | 7 |
| PO | P0A0P9 | Lactose phosphotransferase system repressor | lacR | 0.03504 | -0.595726 | 7 |
| PO | P0C000 | Response regulator ArlR | arlR | 0.00967971 | -0.705247 | 7 |
| PO | P56740 | Dihydroneopterin aldolase | folB | 0.0200909 | -0.782757 | 7 |
| PO | P64375 | Acetoin utilization protein AcuC | acuC | 0.0436707 | -1.15758 | 7 |
| PO | P69775 | Protein map | map | 0.0476345 | -2.80859 | 7 |
| PO | P69775 | Protein map | map | 0.0435823 | -1.63461 | 7 |
| PO | Q2FH01 | L-threonine dehydratase catabolic TdcB | tdcB | 0.0341662 | -1.69075 | 7 |
| PO | Q2FH83 | Uncharacterized protein SAUSA300_1248 | MW1239 | 0.0330385 | -0.824591 | 7 |
| PO | Q2G0P7 | Protein-arginine kinase activator protein | mcsA | 0.036055 | -0.731599 | 7 |
| PO | Q2G2X2 | Glycerol-3-phosphate cytidylyltransferase | tarD | 0.0331191 | -0.582589 | 7 |
| PO | Q2YT09 | Endoribonuclease YbeY | ybeY | 0.0330385 | -1.06235 | 7 |
| PO | Q2YT09 | Endoribonuclease YbeY | ybeY | 0.0114383 | -1.73623 | 7 |
| PO | Q2YUL0 | Aminopyrimidine aminohydrolase | tenA | 0.024596 | -0.627481 | 7 |
| PO | Q2YVZ4 | Immunoglobulin-binding protein Sbi | sbi | 0.00967971 | -2.06208 | 7 |
| PO | Q2YXE6 | Uncharacterized N-acetyltransferase SAB1040c | MW1059 | 0.0330385 | -1.18774 | 7 |
| PO | Q5HF07 | Riboflavin biosynthesis protein RibBA | ribBA | 0.0363645 | -0.978388 | 7 |
| PO | Q6GE13 | Gamma-hemolysin component C | hlgC | 0.0363645 | -1.87276 | 7 |
| PO | Q6GE15 | Immunoglobulin-binding protein Sbi | sbi | 0.00920777 | -2.34957 | 7 |
| PO | Q6GFB8 | Protein map | map | 0.0331191 | -3.10666 | 7 |
| PO | Q6GG12 | Isocitrate dehydrogenase [NADP] | icd | 0.0256709 | -0.846252 | 7 |
| PO | Q6GHJ9 | UPF0122 protein SAR1212 | SAR1212 | 0.00865474 | -1.05743 | 7 |
| PO | Q6GHS9 | Fibrinogen-binding protein | fib | 0.0222211 | -0.944165 | 7 |
| PO | Q6GII7 | 3-dehydroquinate dehydratase | aroD | 0.0183455 | -0.967941 | 7 |
| PO | Q6GJM1 | Probable inorganic carbon transporter subunit DabA | dabA | 0.0223921 | -0.816637 | 7 |
| PO | Q6GKC2 | N-acetyl-gamma-glutamyl-phosphate reductase | argC | 0.0435823 | -1.12151 | 7 |
| C | A0A0H2WWP1 | UDP-N-acetylmuramoyl-tripeptide--D-alanyl-D-alanine ligase | murF | 0.0476308 | 0.582904 | 2 |
| C | Q2FYJ6 | Extracellular matrix-binding protein ebh | ebh | 0.0476308 | 1.56177 | 2 |
| C | Q2FYJ6 | Extracellular matrix-binding protein ebh | ebh | 0.0403611 | 1.56756 | 2 |
| C | A5ISW6 | Extracellular matrix-binding protein ebh | ebh | 0.0218175 | 1.20188 | 2 |
| C | A5ISW6 | Extracellular matrix-binding protein ebh | ebh | 0.046177 | 1.573 | 2 |
| C | A5ITM3 | Acetyl-coenzyme A carboxylase carboxyl transferase subunit alpha | accA | 0.0323944 | 0.79049 | 2 |
| C | Q2FWG4 | Membrane protein insertase YidC | yidC | 0.0111404 | 0.664175 | 2 |
| C | A6QG68 | Ornithine carbamoyltransferase | argF | 0.0233548 | 1.70377 | 2 |
| C | P60747 | Foldase protein PrsA | prsA | 0.0403611 | 0.985066 | 2 |
| C | A5IU67 | Glutamyl-tRNA(Gln) amidotransferase subunit A | gatA | 0.0333516 | 0.886941 | 2 |
| C | A6U1G3 | UPF0154 protein SaurJH1_1431 | MW1230 | 0.0235797 | 1.49835 | 2 |
| C | P0A031 | Cell division protein FtsZ | ftsZ | 0.029907 | 0.62657 | 2 |
| C | P63871 | Cysteine synthase | cysK | 0.0041263 | 0.760729 | 2 |
| C | Q2FK71 | N-acetylmuramic acid 6-phosphate etherase | murQ | 0.0400252 | 0.8183 | 2 |
| C | Q2FV99 | Sortase A | srtA | 0.0228647 | 0.83851 | 2 |
| C | Q2FYJ6 | Extracellular matrix-binding protein ebh | ebh | 0.0403611 | 1.24092 | 2 |
| C | Q2G131 | Efem/EfeO family lipoprotein | MW0319 | 0.0218175 | 1.43639 | 2 |
| C | Q2FV64 | Copper-exporting P-type ATPase | copA | 0.0400252 | 1.15417 | 2 |
| C | Q6GE63 | Uncharacterized lipoprotein SAR2457 | SAR2457 | 0.000339147 | 1.37083 | 2 |
| C | Q2YTZ6 | Foldase protein PrsA | prsA | 0.0476308 | 1.07434 | 2 |
| C | Q6GGX3 | Extracellular matrix-binding protein ebh | ebh | 0.0403611 | 1.56739 | 2 |
| C | Q6GGX3 | Extracellular matrix-binding protein ebh | ebh | 0.0403611 | 1.58507 | 2 |
| C | Q6GI62 | Serine protease HtrA-like | SAR0992 | 0.0165297 | 0.846499 | 2 |
| C | A0A0H2WWB5 | o-succinylbenzoate synthase | menC | 0.0476308 | -0.63458 | 7 |
| C | A5IT65 | Aminomethyltransferase | gcvT | 0.029907 | -0.646877 | 7 |
| C | A5ITB0 | Heat-inducible transcription repressor HrcA | hrcA | 0.0233548 | -0.665599 | 7 |
| C | P20831 | DNA gyrase subunit A | gyrA | 0.0493346 | -0.61395 | 7 |
| C | P64180 | Glyceraldehyde-3-phosphate dehydrogenase 2 | gapA2 | 0.0218175 | -1.13555 | 7 |
| C | P65421 | Probable malate:quinone oxidoreductase 1 | mqo1 | 0.0400252 | -0.839584 | 7 |
| C | Q2G0I9 | Octanoyl-[GcvH]:protein N-octanoyltransferase | lipL | 0.0218175 | -0.852447 | 7 |
| C | Q2G2X2 | Glycerol-3-phosphate cytidylyltransferase | tarD | 0.0366022 | -0.75242 | 7 |
| C | Q6GDS9 | Fructose-1,6-bisphosphatase class 3 | fbp | 0.00784949 | -0.760094 | 7 |
| C | Q6GGG3 | Probable glycine dehydrogenase (decarboxylating) subunit 1 | gcvPA | 0.0228647 | -0.795113 | 7 |
| C | Q6GII7 | 3-dehydroquinate dehydratase | aroD | 0.0299327 | -0.917829 | 7 |
| PP | A0A0H3JR16 | Lipoate--protein ligase 1 | SAV1028 | 0.0394846 | 0.606703 | 2 |
| PP | A5INQ6 | Large ribosomal subunit protein bL9 | rplI | 0.0259989 | 1.45542 | 2 |
| PP | A5IP58 | PTS system glucose-specific EIICBA component | ptsG | 0.0024815 | 0.79774 | 2 |
| PP | A5IQE1 | Coproheme decarboxylase | chdC | 0.0182819 | 0.979923 | 2 |
| PP | A5IRZ2 | Divalent metal cation transporter MntH | mntH | 0.00470453 | 1.02069 | 2 |
| PP | A5IS03 | Protoheme IX farnesyltransferase | ctaB | 0.011932 | 1.0372 | 2 |
| PP | A5IS81 | Lipoprotein signal peptidase | lspA | 0.0207004 | 0.896383 | 2 |
| PP | A5ISC3 | Small ribosomal subunit protein bS16 | rpsP | 0.0253255 | 1.15776 | 2 |
| PP | A5ISN6 | Glycerol-3-phosphate acyltransferase | plsY | 0.032769 | 1.11199 | 2 |
| PP | A5ISW6 | Extracellular matrix-binding protein ebh | ebh | 0.00618639 | 1.5404 | 2 |
| PP | A5ISW6 | Extracellular matrix-binding protein ebh | ebh | 0.0077481 | 1.39212 | 2 |
| PP | A5ISW6 | Extracellular matrix-binding protein ebh | ebh | 0.00524381 | 1.06762 | 2 |
| PP | A5ISW6 | Extracellular matrix-binding protein ebh | ebh | 0.011932 | 1.64529 | 2 |
| PP | A5IT03 | GTPase Der | der | 0.0271383 | 0.760756 | 2 |
| PP | A5ITG9 | Large ribosomal subunit protein bL27 | rpmA | 0.032916 | 0.658993 | 2 |
| PP | A5ITM3 | Acetyl-coenzyme A carboxylase carboxyl transferase subunit alpha | accA | 0.0119965 | 0.703804 | 2 |
| PP | A5IU79 | UPF0316 protein SaurJH9_1967 | MW1852 | 0.0125537 | 0.878592 | 2 |
| PP | A5IUN6 | Membrane protein insertase YidC | yidC | 0.00270078 | 1.15294 | 2 |
| PP | A5IUP4 | 3-hydroxyacyl-[acyl-carrier-protein] dehydratase FabZ | fabZ | 0.032769 | 1.36111 | 2 |
| PP | A5IV07 | Large ribosomal subunit protein bL17 | rplQ | 0.0077481 | 0.739688 | 2 |
| PP | A5IV15 | Large ribosomal subunit protein uL15 | rplO | 0.0261606 | 0.863828 | 2 |
| PP | A5IV16 | Large ribosomal subunit protein uL30 | rpmD | 0.0299217 | 1.05235 | 2 |
| PP | A5IV30 | Small ribosomal subunit protein uS19 | rpsS | 0.0281066 | 1.10646 | 2 |
| PP | A5IVG9 | Probable nitrate transporter NarT | narT | 0.00276241 | 1.04689 | 2 |
| PP | A6QG68 | Ornithine carbamoyltransferase | argF | 0.0077481 | 1.75475 | 2 |
| PP | A6QGG8 | Translation initiation factor IF-2 | infB | 0.00629474 | 1.40995 | 2 |
| PP | A6QI23 | Foldase protein PrsA | prsA | 0.0077481 | 1.61105 | 2 |
| PP | A6U1G3 | UPF0154 protein SaurJH1_1431 | MW1230 | 0.0145269 | 1.87315 | 2 |
| PP | A7WYS8 | Lysine--tRNA ligase | lysS | 0.0388073 | 0.700645 | 2 |
| PP | A8Z324 | Small ribosomal subunit protein uS9 | rpsI | 0.0303319 | 0.768513 | 2 |
| PP | A8Z3Z7 | UDP-N-acetylglucosamine--N-acetylmuramyl-(pentapeptide) pyrophosphoryl-undecaprenol N-acetylglucosamine transferase | murG | 0.0125769 | 0.889369 | 2 |
| PP | A8Z3Z7 | UDP-N-acetylglucosamine--N-acetylmuramyl-(pentapeptide) pyrophosphoryl-undecaprenol N-acetylglucosamine transferase | murG | 0.0087892 | 0.710283 | 2 |
| PP | O08387 | Protein translocase subunit SecY | secY | 0.0269818 | 0.786014 | 2 |
| PP | O32421 | Probable cell wall amidase LytH | lytH | 0.00456515 | 0.998642 | 2 |
| PP | P0C0Z1 | Response regulator protein VraR | vraR | 0.0459562 | 1.4293 | 2 |
| PP | P11162 | PTS system lactose-specific EIICB component | lacE | 0.00270078 | 0.969126 | 2 |
| PP | P14503 | Uncharacterized 27.7 kDa protein |  | 0.00270078 | 0.607735 | 2 |
| PP | P60074 | UPF0291 protein SAV1341 | MW1228 | 0.0282468 | 1.36134 | 2 |
| PP | P60875 | Uncharacterized protein SAV2627.1 | MW2548 | 0.0204744 | 1.02699 | 2 |
| PP | P63332 | Putative zinc metalloprotease SAV1262 | MW1145 | 0.00524381 | 0.869758 | 2 |
| PP | P63870 | Cysteine synthase | cysK | 0.0077481 | 0.650518 | 2 |
| PP | P64164 | DNA translocase FtsK | ftsK | 0.0328688 | 0.977111 | 2 |
| PP | P64333 | Delta-aminolevulinic acid dehydratase | hemB | 0.0290024 | 0.633479 | 2 |
| PP | P67592 | Tryptophan--tRNA ligase | trpS | 0.0164353 | 1.01844 | 2 |
| PP | Q2FFZ9 | UPF0478 protein SAUSA300_1685 | MW1682 | 0.0252366 | 0.581449 | 2 |
| PP | Q2FI19 | Probable quinol oxidase subunit 3 | qoxC | 0.0135567 | 1.05516 | 2 |
| PP | Q2FK71 | N-acetylmuramic acid 6-phosphate etherase | murQ | 0.0077481 | 0.729466 | 2 |
| PP | Q2FV54 | O-acetyltransferase OatA | SACOL0978 | 0.032769 | 1.70312 | 2 |
| PP | Q2FV54 | O-acetyltransferase OatA | oatA | 0.0024815 | 1.12008 | 2 |
| PP | Q2FV99 | Sortase A | srtA | 0.000481232 | 1.22684 | 2 |
| PP | Q2FVY9 | HTH-type transcriptional regulator SarV | sarV | 0.0156864 | 0.795898 | 2 |
| PP | Q2FXA5 | Coproporphyrinogen III oxidase | cgoX | 0.0418405 | 0.651 | 2 |
| PP | Q2FXP9 | Translation initiation factor IF-3 | infC | 0.0054109 | 0.92322 | 2 |
| PP | Q2FYJ6 | Extracellular matrix-binding protein ebh | ebh | 0.0100202 | 1.40469 | 2 |
| PP | Q2FZ91 | Cell division protein DivIB | divIB | 0.0388413 | 0.869843 | 2 |
| PP | Q2G026 | Protein translocase subunit SecG | SAOUHSC_00801 | 0.0394846 | 0.675959 | 2 |
| PP | Q2G131 | Efem/EfeO family lipoprotein | MW0319 | 0.00470453 | 1.40839 | 2 |
| PP | Q2YTE1 | Acetyl-coenzyme A carboxylase carboxyl transferase subunit alpha | accA | 0.0132539 | 0.821557 | 2 |
| PP | Q53630 | Elastin-binding protein EbpS | ebpS | 0.032769 | 1.44586 | 2 |
| PP | Q5HF12 | HTH-type transcriptional regulator rot | rot | 0.0388818 | 0.612695 | 2 |
| PP | Q5HFV0 | DNA-binding protein HU | hup | 0.0241767 | 1.25466 | 2 |
| PP | Q5HG57 | Regulatory protein MsrR | msrR | 0.0182819 | 0.725819 | 2 |
| PP | Q5HGH0 | Phosphatidate cytidylyltransferase | cdsA | 0.0315607 | 0.714804 | 2 |
| PP | Q6GE63 | Uncharacterized lipoprotein SAR2457 | SAR2457 | 0.000618479 | 1.66655 | 2 |
| PP | Q6GE74 | Putative hemin transport system permease protein HrtB | hrtB | 0.0472951 | 0.645404 | 2 |
| PP | Q6GEA0 | Lysostaphin resistance protein A | lyrA | 0.0161312 | 1.54905 | 2 |
| PP | Q6GES1 | PTS system mannitol-specific EIICB component | mtlA | 0.0281569 | 1.37081 | 2 |
| PP | Q6GFL5 | Foldase protein PrsA | prsA | 0.0108473 | 1.46059 | 2 |
| PP | Q6GG09 | Pyruvate kinase | pyk | 0.0238692 | 0.766983 | 2 |
| PP | Q6GG25 | Translation initiation factor IF-3 | infC | 0.0132539 | 1.12219 | 2 |
| PP | Q6GG32 | Probable GTP-binding protein EngB | engB | 0.0132539 | 0.892746 | 2 |
| PP | Q6GGX3 | Extracellular matrix-binding protein ebh | ebh | 0.00812455 | 1.51884 | 2 |
| PP | Q6GGX3 | Extracellular matrix-binding protein ebh | ebh | 0.00524381 | 1.61691 | 2 |
| PP | Q6GGZ0 | UDP-N-acetylglucosamine--N-acetylmuramyl-(pentapeptide) pyrophosphoryl-undecaprenol N-acetylglucosamine transferase | murG | 0.0433776 | 0.835845 | 2 |
| PP | Q6GH31 | Aminoacyltransferase FemA | femA | 0.0388073 | 0.668359 | 2 |
| PP | Q6GHD4 | Aerobic glycerol-3-phosphate dehydrogenase | glpD | 0.0135567 | 0.739183 | 2 |
| PP | Q6GI31 | Bifunctional autolysin | atl | 0.0124956 | 1.21712 | 2 |
| PP | Q6GI62 | Serine protease HtrA-like | SAR0992 | 0.00524381 | 1.11463 | 2 |
| PP | Q6GIN0 | Phosphatidylglycerol--prolipoprotein diacylglyceryl transferase | lgt | 0.0301391 | 0.829889 | 2 |
| PP | Q6GJC5 | DNA-directed RNA polymerase subunit beta' | rpoC | 0.0388073 | 0.603586 | 2 |
| PP | A0A0H2WWB5 | o-succinylbenzoate synthase | menC | 0.0161312 | -0.671106 | 7 |
| PP | A0A0H2XHV5 | 6-phospho-N-acetylmuramidase | mupG | 0.0328688 | -2.18209 | 7 |
| PP | A5IPX0 | GMP synthase [glutamine-hydrolyzing] | guaA | 0.00272951 | -0.704561 | 7 |
| PP | A5IQ06 | Probable inorganic carbon transporter subunit DabA | dabA | 0.00470453 | -0.615426 | 7 |
| PP | A5IQ73 | Pyridoxal 5'-phosphate synthase subunit PdxT | pdxT | 0.0077481 | -1.02867 | 7 |
| PP | A5IQR6 | 7-cyano-7-deazaguanine synthase | queC | 0.0182819 | -1.81683 | 7 |
| PP | A5IRB2 | D-alanyl carrier protein | dltC | 0.00270078 | -1.19909 | 7 |
| PP | A5IRJ1 | Putative phosphoesterase SaurJH9_1013 | MW0896 | 0.00524381 | -1.93269 | 7 |
| PP | A5IRZ9 | UPF0358 protein SaurJH9_1172 | MW0995 | 0.00270078 | -2.23685 | 7 |
| PP | A5IS36 | Glutamate racemase | murI | 0.0259283 | -1.1729 | 7 |
| PP | A5ISD0 | Succinate--CoA ligase [ADP-forming] subunit beta | sucC | 0.00456515 | -0.952535 | 7 |
| PP | A5ISV8 | Thymidylate synthase | thyA | 0.0109795 | -0.818484 | 7 |
| PP | A5IT65 | Aminomethyltransferase | gcvT | 0.00482923 | -0.60294 | 7 |
| PP | A5ITB0 | Heat-inducible transcription repressor HrcA | hrcA | 0.0024054 | -0.746774 | 7 |
| PP | A5ITE0 | UPF0473 protein SaurJH9_1672 | MW1565 | 0.0207004 | -1.91751 | 7 |
| PP | A5ITW2 | 2-succinylbenzoate--CoA ligase | menE | 0.0077481 | -0.694311 | 7 |
| PP | A5IU61 | DNA polymerase IV | dinB | 0.0316536 | -0.791473 | 7 |
| PP | A5IU73 | Heptaprenylglyceryl phosphate synthase | pcrB | 0.0110305 | -0.696642 | 7 |
| PP | A5IUL7 | Holo-[acyl-carrier-protein] synthase | acpS | 0.0248926 | -0.66991 | 7 |
| PP | A5IUS3 | Probable DNA-directed RNA polymerase subunit delta | rpoE | 0.0315607 | -1.43366 | 7 |
| PP | A5IUS9 | S-ribosylhomocysteine lyase | luxS | 0.00270078 | -0.609534 | 7 |
| PP | A5IV33 | Large ribosomal subunit protein uL4 | rplD | 0.0415881 | -0.632394 | 7 |
| PP | A5IV69 | Urease subunit gamma | ureA | 0.0174237 | -0.699437 | 7 |
| PP | A5IVB2 | Imidazolonepropionase | hutI | 0.0373422 | -0.942215 | 7 |
| PP | A5IW37 | Oxygen-dependent choline dehydrogenase | betA | 0.0323963 | -0.653593 | 7 |
| PP | A5IW58 | HTH-type transcriptional regulator ArcR | arcR | 0.0271383 | -1.47859 | 7 |
| PP | A6QDV0 | Putative N-acetylmannosamine-6-phosphate 2-epimerase | nanE | 0.0323963 | -0.979862 | 7 |
| PP | A6QED4 | Recombination protein RecR | recR | 0.0252366 | -1.23624 | 7 |
| PP | A6QED4 | Recombination protein RecR | recR | 0.0142908 | -1.52441 | 7 |
| PP | A6QEK4 | Ribulokinase | araB | 0.00456515 | -1.37922 | 7 |
| PP | A6QFH8 | ATP-dependent helicase/nuclease subunit A | addA | 0.0468254 | -0.757129 | 7 |
| PP | A6QFL5 | UPF0738 protein NWMN_0875 | MW0886 | 0.0132539 | -1.04254 | 7 |
| PP | A6QHA9 | DNA repair protein RecO | recO | 0.00272951 | -0.693806 | 7 |
| PP | A7WXH5 | N-acetyl-gamma-glutamyl-phosphate reductase | argC | 0.0315607 | -0.813239 | 7 |
| PP | A9JX08 | Phenol-soluble modulin alpha 4 peptide | psmA4 | 0.0265958 | -4.56096 | 7 |
| PP | P09978 | Phospholipase C | hlb | 0.0207004 | -1.07771 | 7 |
| PP | P0A022 | Beta sliding clamp | dnaN | 0.0135567 | -0.651239 | 7 |
| PP | P0A071 | Gamma-hemolysin component A | hlgA | 0.0368421 | -1.52565 | 7 |
| PP | P0A071 | Gamma-hemolysin component A | hlgA | 0.0314705 | -1.56291 | 7 |
| PP | P0A071 | Gamma-hemolysin component A | hlgA | 0.0172064 | -1.02361 | 7 |
| PP | P0A0I5 | Accessory gene regulator protein A | agrA | 0.0077481 | -2.88569 | 7 |
| PP | P0A0P9 | Lactose phosphotransferase system repressor | lacR | 0.0312535 | -0.656215 | 7 |
| PP | P0C000 | Response regulator ArlR | arlR | 0.00456515 | -0.913995 | 7 |
| PP | P0C048 | Type VII secretion system protein EssC | essC | 0.0443732 | -0.864928 | 7 |
| PP | P56740 | Dihydroneopterin aldolase | folB | 0.00524381 | -0.922338 | 7 |
| PP | P60070 | Anti-sigma-B factor antagonist | rsbV | 0.0125743 | -1.18075 | 7 |
| PP | P61084 | Deoxyribose-phosphate aldolase 2 | deoC1 | 0.0305069 | -0.82836 | 7 |
| PP | P61084 | Deoxyribose-phosphate aldolase 2 | deoC1 | 0.0315607 | -0.952088 | 7 |
| PP | P64180 | Glyceraldehyde-3-phosphate dehydrogenase 2 | gapA2 | 0.00270402 | -0.82825 | 7 |
| PP | P64375 | Acetoin utilization protein AcuC | acuC | 0.0491526 | -1.12777 | 7 |
| PP | P65421 | Probable malate:quinone oxidoreductase 1 | mqo1 | 0.00629474 | -0.613931 | 7 |
| PP | P69775 | Protein map | map | 0.0388073 | -2.78352 | 7 |
| PP | P69775 | Protein map | map | 0.0207004 | -1.35761 | 7 |
| PP | Q2FFH4 | Uncharacterized protein SAUSA300_1902 | MW1862 | 0.00524381 | -0.702386 | 7 |
| PP | Q2FH83 | Uncharacterized protein SAUSA300_1248 | MW1239 | 0.0164353 | -1.39479 | 7 |
| PP | Q2FIC1 | Putative peptidyl-prolyl cis-trans isomerase | MW0836 | 0.0135567 | -0.885714 | 7 |
| PP | Q2FK43 | Pyruvate formate-lyase-activating enzyme | pflA | 0.00524381 | -0.77977 | 7 |
| PP | Q2FUW3 | Accessory Sec system protein Asp1 | asp1 | 0.0207004 | -0.918637 | 7 |
| PP | Q2FUW5 | Accessory Sec system protein Asp3 | asp3 | 0.032769 | -0.72877 | 7 |
| PP | Q2FYJ2 | Alanine dehydrogenase 1 | ald1 | 0.0077481 | -0.832273 | 7 |
| PP | Q2G0I9 | Octanoyl-[GcvH]:protein N-octanoyltransferase | lipL | 0.00270078 | -1.04944 | 7 |
| PP | Q2G0P7 | Protein-arginine kinase activator protein | mcsA | 0.0029208 | -1.22167 | 7 |
| PP | Q2G2J8 | Uncharacterized protein SAOUHSC_01413 | MW1299 | 0.0164353 | -0.799362 | 7 |
| PP | Q2G2X2 | Glycerol-3-phosphate cytidylyltransferase | tarD | 0.0142979 | -0.739878 | 7 |
| PP | Q2YT09 | Endoribonuclease YbeY | ybeY | 0.00401438 | -1.51758 | 7 |
| PP | Q2YT09 | Endoribonuclease YbeY | ybeY | 0.0115537 | -1.30946 | 7 |
| PP | Q2YUL2 | Thiamine-phosphate synthase | thiE | 0.0149722 | -1.46152 | 7 |
| PP | Q2YWR1 | Acid sugar phosphatase | nagD | 0.0302833 | -4.64426 | 7 |
| PP | Q2YXE6 | Uncharacterized N-acetyltransferase SAB1040c | MW1059 | 0.00904017 | -1.87541 | 7 |
| PP | Q2YZB4 | Histidinol dehydrogenase | hisD | 0.0328367 | -0.593863 | 7 |
| PP | Q53599 | Protein map | map | 0.0376854 | -2.07345 | 7 |
| PP | Q5HES4 | Fumarate hydratase class II | fumC | 0.0182819 | -0.719859 | 7 |
| PP | Q5HIS8 | Single-stranded DNA-binding protein 1 | ssb;ssb2 | 0.0252366 | -0.648814 | 7 |
| PP | Q6GCL1 | Transcriptional regulatory protein LytR | lytR | 0.0204744 | -0.794837 | 7 |
| PP | Q6GE12 | Gamma-hemolysin component B | hlgB | 0.0253255 | -1.18646 | 7 |
| PP | Q6GE13 | Gamma-hemolysin component C | hlgC | 0.0259283 | -2.00786 | 7 |
| PP | Q6GE15 | Immunoglobulin-binding protein Sbi | sbi | 0.0348855 | -1.43895 | 7 |
| PP | Q6GE66 | Probable malate:quinone oxidoreductase 1 | mqo1 | 0.0077481 | -1.38043 | 7 |
| PP | Q6GE99 | Ribose-5-phosphate isomerase A | rpiA | 0.00132308 | -0.79702 | 7 |
| PP | Q6GEW1 | UPF0340 protein SAR2202 | SAR2202 | 0.00665371 | -0.895037 | 7 |
| PP | Q6GFK5 | Fumarate hydratase class II | fumC | 0.00272951 | -1.21573 | 7 |
| PP | Q6GFX3 | Acetoin utilization protein AcuC | acuC | 0.0135567 | -1.14252 | 7 |
| PP | Q6GG04 | DNA polymerase III subunit alpha | dnaE | 0.0132539 | -0.801667 | 7 |
| PP | Q6GG12 | Isocitrate dehydrogenase [NADP] | icd | 0.00713548 | -1.00923 | 7 |
| PP | Q6GGH6 | Exodeoxyribonuclease 7 small subunit | xseB | 0.0432049 | -1.61663 | 7 |
| PP | Q6GGK4 | Segregation and condensation protein B | scpB | 0.0446049 | -0.766719 | 7 |
| PP | Q6GGW9 | Alanine dehydrogenase 1 | ald1 | 0.000618479 | -1.21351 | 7 |
| PP | Q6GHJ9 | UPF0122 protein SAR1212 | SAR1212 | 0.00272951 | -1.6194 | 7 |
| PP | Q6GI10 | Phosphoribosylamine--glycine ligase | purD | 0.00482923 | -0.793123 | 7 |
| PP | Q6GII7 | 3-dehydroquinate dehydratase | aroD | 0.00270078 | -1.19601 | 7 |
| PP | Q6GJM1 | Probable inorganic carbon transporter subunit DabA | dabA | 0.00152085 | -1.0795 | 7 |
| PP | Q6GKC2 | N-acetyl-gamma-glutamyl-phosphate reductase | argC | 0.0257429 | -1.25636 | 7 |
| PP | Q6GKM5 | Uncharacterized chromosomal cassette SCCmec type IVc protein CR006 | CR006 | 0.0292655 | -1.07928 | 7 |

**
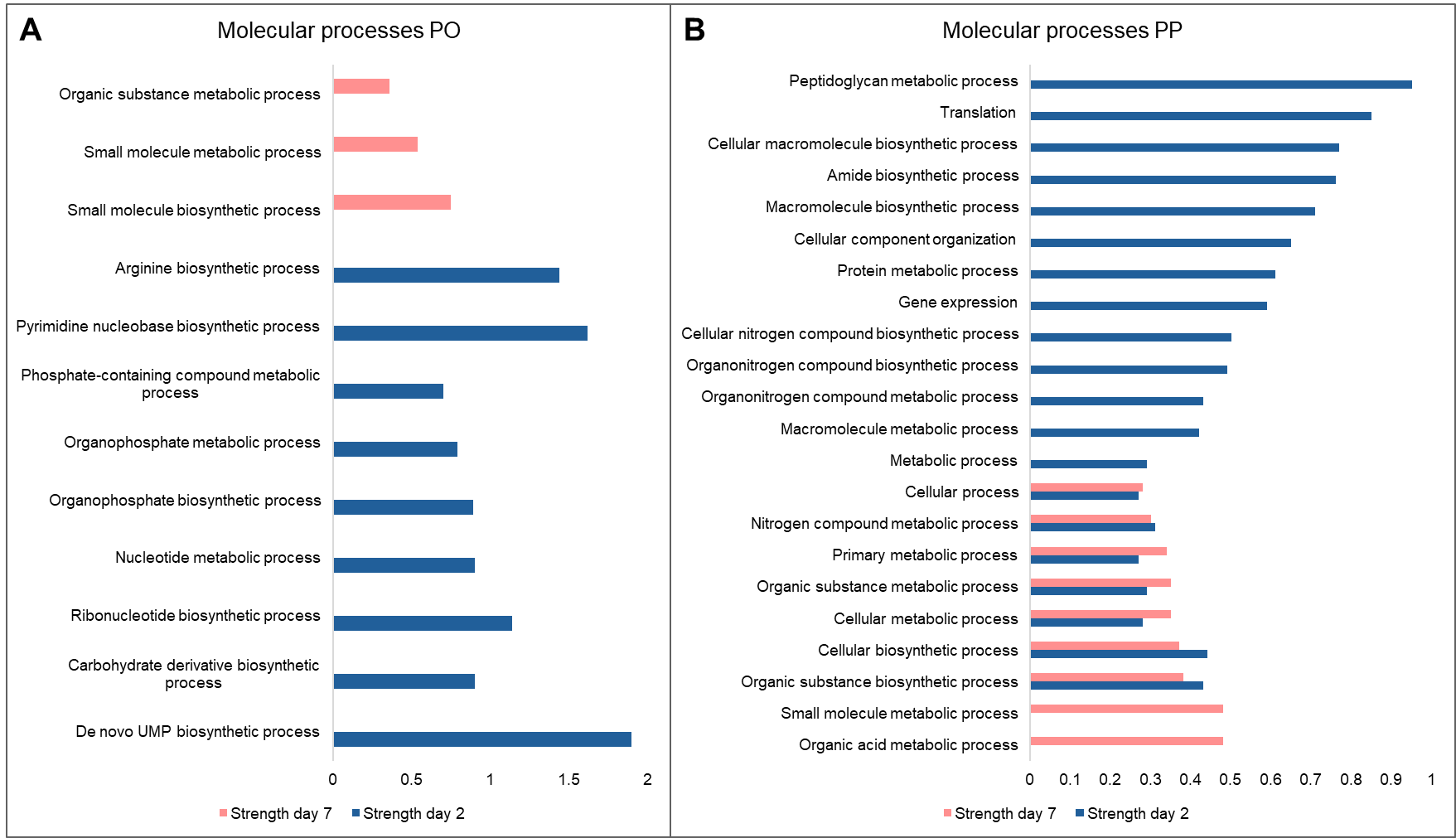
**

**Figure S4. Full list of molecular and cellular functions upregulated per biofilm growth state grown on Polished and plasma pore disks.** GO molecular and cellular functions are upregulated in the mature biofilm and are shown in pink. Blue shows GO molecular and cellular functions upregulated in the immature biofilm.

**
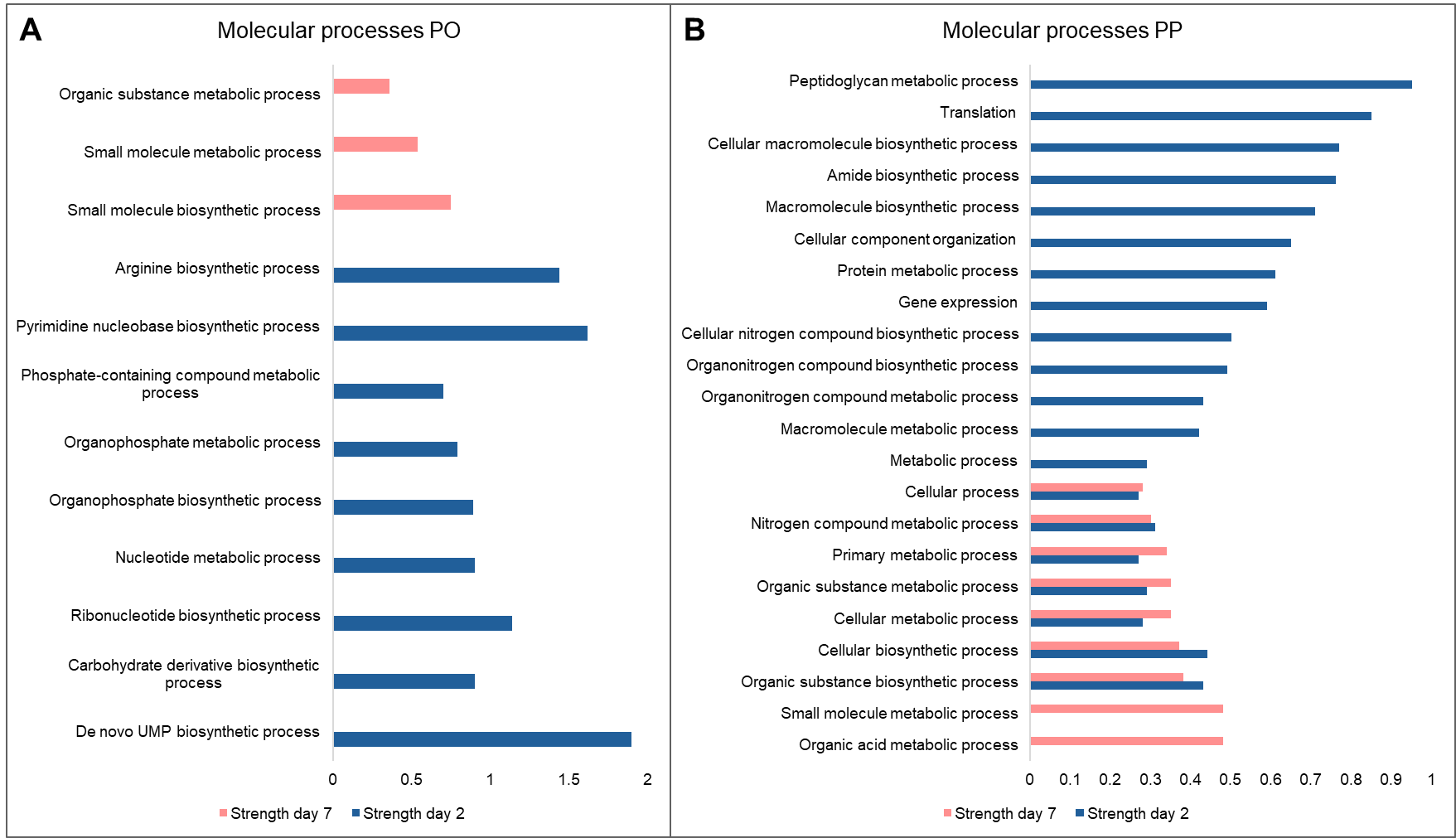
**

**Table S3: Deregulated proteins in S.aureus grown on three titanium surfaces with different roughness.** The table lists the deregulated proteins, cellular components,and molecules found in the literature that showed potential inhibition of the protein

| Protein ID | Protein Description | Genes name | Cluster | Description | Cellular component component | Inhibition | Reference |
| --- | --- | --- | --- | --- | --- | --- | --- |
| Q2YT97 | D-aminoacyl-tRNA deacetylase | dtd | Olive | D-aminoacyl-tRNA deacylase is an enzyme that cleaves mischarged D-aminoacyl-tRNA into free tRNAs and D-amino acids | Cytoplasm | - |  |
| A6QEL9 | GTP cyclohydrolase FolE2 | folE2 | Olive | Converts GTP to 7,8-dihydroneopterin triphosphate. | - | - |  |
| Q6GGY3 | Peptide methionine sulfoxide reductase MsrA 2 | msrA2 | Olive | Peptide methionine sulfoxide reductase MsrA 2 repairs enzymes for proteins inactivated by oxidation. | - | - |  |
| A5IW58 | HTH-type transcriptional regulator ArcR | arcR | Olive | positively regulates the expression of the arcABDCR operon under anaerobic conditions, thus playing an essential role in arginine catabolism. May also control the expression of genes encoding proteins involved in anaerobic metabolism. Can bind cyclic AMP (By similarity) | - | - |  |
| Q6GIR8 | Histidinol-phosphate aminotransferase | hisC | Olive | Histidinol-phosphate aminotransferase is involved in the biosynthesis of the amino acid histidine. | Chromosome | Heraclenol | ^1,2,3,4^ |
| Q6GDP1 | Copper-exporting P-type ATPase | copA | Purple | Involved in copper export. | plasma membrane |  |  |
| Q6GI31 | Bifunctional autolysin | atl | Purple | Alt is a important surface protein of *S.aureus*. It is a peptidoglycan involved in cell wall degradation and separation during cell division. | extracellular region | ZJ-2 | ^5, 6^ |
| A5ITA3 | Small ribosomal subunit protein bS21 | rpsU | Purple | This protein is a small ribosomal subunit protein. | Ribosome | - |  |
| A6U1G3 | UPF0154 protein SaurJH1_1431 | MW1230 | Purple | - | plasma membrane |  |  |
| Q6GHI8 | Probable cell wall hydrolase LytN | lytN | Purple | LytN has amidase and peptidase activities on staphylococcus peptidoglycan | extracellular region | Magnolol | ^7^ |
| Q6GKD8 | Putative aldehyde dehydrogenase AldA | aldA | Pink | - | Chromosome | - |  |
| Q6GFR5 | Phosphoenolpyruvate carboxykinase (ATP) | pckA | Pink | It is involved in gluconeogenesis, a metabolic pathway that results in the biosynthesis of glucose. | Cytosol | - |  |
| P67417 | Urocanate hydratase | hutU | Pink | Catalyzes the conversion of urocanate to 4-imidazoline-5-propionate, an intermediate in the metabolism of histidine | Cytoplasm | - |  |
| P64180 | Glyceraldehyde-3-phosphate dehydrogenase 2 | gapA2 | Pink | Catalyzes the oxidative phosphorylation of glyceraldehyde 3-phosphate (G3P) to 1,3-bisphosphoglycerate (BPG) using the cofactor NAD. | Cytosol | Iodine-containing complex | ^8^ |
| A5IVY0 | 1-pyrroline-5-carboxylate dehydrogenase | rocA | Pink | Degradation of Amino-acids | - | - |  |
| Q2YTF6 | Formate-tetrahydrofolate ligase | fhs | Pink | - | - | Antimicrobial peptides CGS19 and CGS20 | ^9^ |
| Q6GGG3 | Probable glycine dehydrogenase (decarboxylating) subunit 1 | gcvPA | Pink | The glycine cleavage system catalyzes the degradation of glycine. | - | - |  |
| A5IT65 | Aminomethyltransferase | gcvT | Pink | The glycine cleavage system catalyzes the degradation of glycine. | Cytosol | - |  |
| Q6GGZ6 | Dihydrolipoyllysine-residue succinyltransferase component of 2-oxoglutarate dehydrogenase complex | odhB | Pink | Helps in the catalysis the second step in the conversion of 2-oxoglutarate to succinyl-CoA and CO2. | Cytosol | TCA cycle inhibition | ^10,11^ |
| P65421 | Probable malate: quinone oxidoreductase 1 | mqo1 | Pink | Involved in the carbohydrate metabolism; TCA cycle | - | TCA cycle inhibition | ^10,11^ |
| Q6GHI9 | Succinate--CoA ligase [ADP-forming] subunit alpha | sucD | Pink | Succinyl-CoA synthetase functions in the citric acid cycle (TCA). | Cytosol | TCA cycle inhibition | ^10,11^ |
| Q2FH26 | Dihydrolipoyllysine-residue succinyltransferase component of 2-oxoglutarate dehydrogenase complex | odhB | Pink | Catalyzes the second step in the conversion of 2-oxoglutarate to succinyl-CoA and CO2 in the TCA cycle | Cytosol | TCA cycle inhibition | ^10,11^ |
| A6QGW6 | 2-oxoglutarate dehydrogenase E1 component | odhA | Pink | Involved in the first step in converting 2-oxoglutarate to succinyl-CoA and CO2. Engaged in the TCA cycle | Cytosol | TCA cycle inhibition | ^10,11^ |
| A5ISD0 | Succinate--CoA ligase [ADP-forming] subunit beta | sucC | Pink | Succinyl-CoA synthetase functions in the citric acid cycle (TCA), coupling the hydrolysis of succinyl-CoA to the synthesis of either ATP or GTP | Cytosol | TCA cycle inhibition | ^10,11^ |
| P0A071 | Gamma-hemolysin component A | hlgA | Blue | A yoxin that seems to act by forming pores in the cell membrane. | extracellular region | - |  |
| A5IQ73 | Pyridoxal 5'-phosphate synthase subunit PdxT | pdxT | Blue | Catalyzes the hydrolysis of glutamine to glutamate as part of the biosynthesis of pyridoxal 5'-phosphate. | - | - |  |

**References**

1 Han, B., Wang, H. & Niu, X. A natural inhibitor of diapophytoene desaturase attenuates methicillin-resistant Staphylococcus aureus (MRSA) pathogenicity and overcomes drug-resistance. *Br J Pharmacol* (2024). <https://doi.org/10.1111/bph.16377>

2 Henriksen, S. T., Liu, J., Estiu, G., Oltvai, Z. N. & Wiest, O. Identification of novel bacterial histidine biosynthesis inhibitors using docking, ensemble rescoring, and whole-cell assays. *Bioorg Med Chem* **18**, 5148-5156 (2010). <https://doi.org/10.1016/j.bmc.2010.05.060>

3 Kaur, H. *et al.* In Vitro and In Vivo Studies of Heraclenol as a Novel Bacterial Histidine Biosynthesis Inhibitor against Invasive and Biofilm-Forming Uropathogenic Escherichia coli. *Antibiotics (Basel)* **12** (2023). <https://doi.org/10.3390/antibiotics12010110>

4 Widelski, J., Popova, M., Graikou, K., Glowniak, K. & Chinou, I. Coumarins from Angelica lucida L.--antibacterial activities. *Molecules* **14**, 2729-2734 (2009). <https://doi.org/10.3390/molecules14082729>

5 Xie, Y. *et al.* Antibacterial and anti-biofilm activity of diarylureas against Enterococcus faecium by suppressing the gene expression of peptidoglycan hydrolases and adherence. *Front Microbiol* **13**, 1071255 (2022). <https://doi.org/10.3389/fmicb.2022.1071255>

6 Yang, Y. *et al.* Inhibiting Peptidoglycan Hydrolase Alleviates MRSA Pneumonia Through Autolysin-Mediated MDP-NOD2 Pathway. *Infect Drug Resist* **17**, 1231-1242 (2024). <https://doi.org/10.2147/IDR.S455339>

7 Wang, D. *et al.* Transcriptional and functional analysis of the effects of magnolol: inhibition of autolysis and biofilms in Staphylococcus aureus. *PLoS One* **6**, e26833 (2011). <https://doi.org/10.1371/journal.pone.0026833>

8 Kenesheva, S. T. *et al.* The Effect of Three Complexes of Iodine with Amino Acids on Gene Expression of Model Antibiotic Resistant Microorganisms Escherichia coli ATCC BAA-196 and Staphylococcus aureus ATCC BAA-39. *Microorganisms* **11** (2023). <https://doi.org/10.3390/microorganisms11071705>

9 Li, C. *et al.* Development of Antibacterial Peptides with Membrane Disruption and Folate Pathway Inhibitory Activities against Methicillin-Resistant Staphylococcus aureus. *J Med Chem* **67**, 1044-1060 (2024). <https://doi.org/10.1021/acs.jmedchem.3c01360>

10 Chen, J. *et al.* Metabolomics analysis to evaluate the antibacterial activity of the essential oil from the leaves of Cinnamomum camphora (Linn.) Presl. *J Ethnopharmacol* **253**, 112652 (2020). <https://doi.org/10.1016/j.jep.2020.112652>

11 Xie, L. Y. *et al.* Itaconic acid and dimethyl itaconate exert antibacterial activity in carbon-enriched environments through the TCA cycle. *Biomed Pharmacother* **167**, 115487 (2023). <https://doi.org/10.1016/j.biopha.2023.115487>
